# The differential impacts of environmental air pollution exposure on the risk of placenta previa and placenta accreta in twin pregnancies

**DOI:** 10.3389/fendo.2025.1624480

**Published:** 2025-09-08

**Authors:** Wei-Zhen Tang, Hong Chen, Hong-Yu Xu, Qin-Yu Cai, Niya Zhou, Yi-Fan Zhao, Bo-Yuan Deng, Xu Zhang, Fei Han, Tai-Hang Liu, Zhen Chen

**Affiliations:** ^1^ Department of Obstetrics and Gynecology, Women and Children’s Hospital of Chongqing Medical University, Chongqing, China; ^2^ Department of Biobank, Chongqing Health Center For Women and Children, Chongqing, China; ^3^ School of Basic Medical Sciences, Chongqing Medical University, Chongqing, China; ^4^ The Joint International Research Laboratory of Reproduction and Development, Chongqing Medical University, Chongqing, China

**Keywords:** twin pregnancy, placenta previa, placenta accreta, air pollution, windows of exposure

## Abstract

**Background:**

This study aims to investigate the association between exposure to air pollutants during pregnancy (until placental accreta) in twin pregnancies and the risk of abnormal placental positioning and development.

**Methods:**

This retrospective study included 3,670 pregnant women with twin pregnancies, classified into three groups: no placenta previa or accreta (3,017 cases), placenta previa (119 cases), and placenta accreta without previa (534 cases). Air pollution data (PM2.5, PM10, SO2, NO2, CO, and O3) were collected from 12 monitoring stations in Chongqing. Exposure estimates were calculated using geocoding based on hospital locations and the nearest stations. Multivariate logistic regression and trend regression analyses, adjusted for confounders, assessed the impact of these pollutants on placenta previa and placenta accreta risk during the 3 months preconception, first trimester, and second trimester. Stratified analyses based on maternal characteristics and restricted cubic spline (RCS) analysis were performed. A dual-pollutant model was used to validate the results.

**Results:**

Results showed that none of the six pollutants were significantly associated with placenta previa in either single-pollutant or dual-pollutant models. In the subtypes of placenta previa, NO_2_ was found to be a protective factor for marginal placenta previa only during the 3 months preconception, with no significant associations observed for other pollutants. For placenta accreta, both single-pollutant and dual-pollutant models indicated that exposure to PM_2.5_, PM_10_, SO_2_, and NO_2_ during the 3 months preconception might have a protective effect. In contrast, O_3_ significantly increased the risk of placenta accreta during both the 3 months preconception and the first trimester, regardless of whether it was analyzed in a single-pollutant or dual-pollutant model. Furthermore, the dual-pollutant model revealed that NO_2_ and CO were risk factors for placenta accreta during the second trimester after adjusting for PM_2.5_ and PM_10_. Stratified analyses based on maternal characteristics showed stable associations between the six pollutants and placenta previa across different periods, while the impact of pollutants on placenta accreta varied under different maternal clinical characteristics.

**Conclusion:**

The mechanisms by which air pollutants affect placenta previa and placenta accreta in twin pregnancies are notably different.

## Introduction

The association between ambient air pollution and adverse birth outcomes, such as preterm birth and low birth weight, has garnered widespread attention. However, the specific pathways underlying this relationship have not been fully elucidated. Recent studies have shown that exposure to air pollutants is associated with obstetric complications such as hypertensive disorders of pregnancy (HDP) (1–3), which can lead to preterm birth and intrauterine growth restriction (IUGR). Therefore, HDP may serve as a mediating factor between air pollutant exposure and adverse birth outcomes. These disorders, including preeclampsia and gestational hypertension, are collectively referred to as placenta-mediated pregnancy complications (4), as the placenta, a critical transient organ for fetal development, plays a vital role in fetal growth and is closely related to maternal health. Conversely, placental abnormalities or diseases have been proven to be associated with adverse maternal and fetal outcomes (5). Given this, placental diseases may be a key link between air pollutant exposure and adverse birth outcomes.

Placenta previa and placenta accreta are significant components of placental diseases. Placenta previa, defined as the placenta covering or approaching the internal cervical, is one of the main causes of iatrogenic preterm birth (6). Placenta accreta is characterized by abnormal adherence of placental trophoblasts to the myometrium (7). Both conditions are closely related to maternal and perinatal morbidity and mortality (8–10). Over the past few decades, the frequency of twin pregnancies has increased, primarily due to the delayed childbearing age of women and the widespread use of assisted reproductive technologies (11). Studies have shown that the risk of placental diseases in twin pregnancies is significantly higher. For instance, after adjusting for confounding factors such as smoking, the overall incidence of placenta previa in twin pregnancies is 40% higher than that in singleton pregnancies (12). Additionally, the risk of placenta accreta in twin pregnancies is 2.5 times that of singleton pregnancies (13). Moreover, twin pregnancies are more prone to adverse perinatal outcomes, such as preterm birth, cesarean delivery, and neonatal intensive care unit (NICU) admission (14, 15). Therefore, understanding and controlling risk factors for placental diseases in twin pregnancies is of great significance for clinical practice and research. Although the etiology of placenta previa and placenta accreta is not fully understood, factors such as advanced maternal age, multiparity, smoking, previous cesarean delivery, and prior uterine diseases are considered potential risk factors (6, 16, 17). These factors are associated with endometrial ischemia and/or injury to the endometrium and myometrium, leading to placental diseases. The adverse effects of air pollution on human health, including increased blood viscosity, endothelial dysfunction, and systemic inflammation, may be related to endometrial ischemia and/or injury to the endometrium and myometrium (18). This suggests that exposure to air pollutants may interfere with the normal implantation of the placenta in the upper segment of the uterus (19, 20). Twins provide a unique opportunity to study prenatal environmental influences, as they may be more susceptible to the adverse effects of air pollution during pregnancy than singletons, making the impact of environmental factors more pronounced.

However, few studies in environmental health research have explored the relationship between air pollutants and placenta previa or placenta accreta, and those that exist have primarily focused on singleton pregnancies, with limited attention to twin pregnancies and the most recent developments in the field (20, 21). For example, a study by Takehiro Michikawa et al. on singleton pregnancies found that exposure to particulate matter and ozone during early pregnancy (0–4 weeks) was associated with an increased risk of placenta previa, with odds ratios (OR) of 1.12 (95% CI = 1.01–1.23) and 1.08 (1.00–1.16), respectively. Additionally, exposure to NO_2_ and SO_2_ was directionally associated with an increased risk of placenta previa. Exposure to particulate matter during this period was also associated with abnormal placenta accreta in the absence of placenta previa (OR = 1.33, 95% CI = 1.07–1.66) (20). The aim of this study is to investigate the association between exposure to air pollutants during pregnancy (up to the stage of placenta accreta) in twin pregnancies and the risk of placenta previa and placenta accreta. Air pollution data for this study were collected from 12 monitoring stations located across Chongqing, covering the period from December 2016 to December 2022. This data will provide important scientific evidence for understanding the potential mechanisms and prevention strategies for placental diseases in twin pregnancies.

## Materials and methods

### Ethics approval and consent to participate

This study was approved by the Women and Children’s Hospital of Chongqing Medical University (ID: 2024025). As a retrospective study, the requirement for informed consent was waived by the ethics committee, given the use of anonymized data and the study’s observational nature.

This study in accordance with Declaration of Helsinki. All methods were carried out in accordance with the relevant guidelines and regulations. The participants’ legal guardian or next of kin provided written informed consent for this study.

### Study design

**Figure 1:**
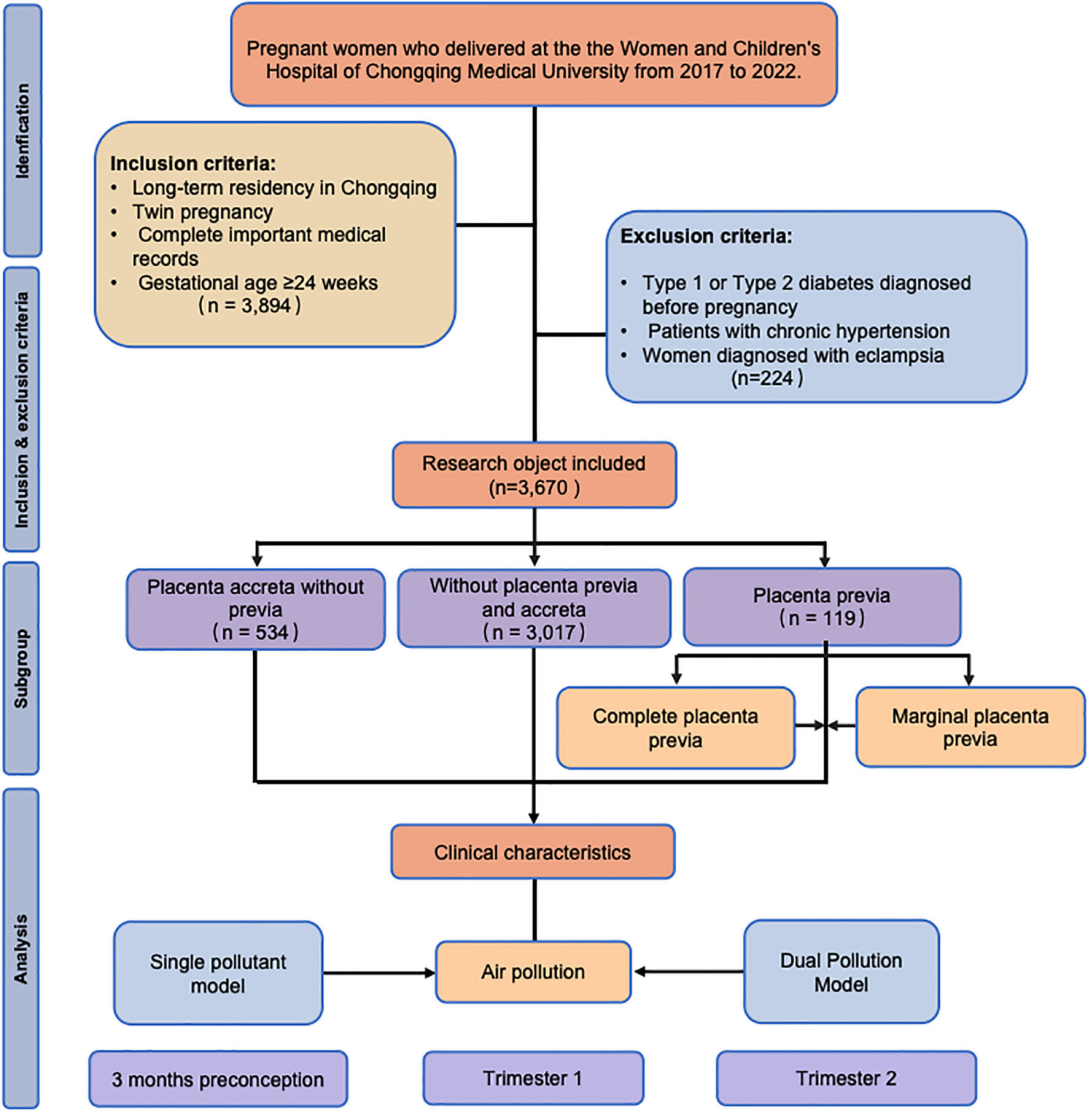
Flowchart of this retrospective cohort study.

This retrospective cohort study included pregnant women who delivered at the Women and Children’s Hospital of Chongqing Medical University from January 2017 to December 2022. The inclusion criteria were as follows: (i) long-term residency in Chongqing; (ii) twin pregnancies; (iii) complete important medical records; and (iv) gestational age ≥24 weeks. The operational definition of the criterion of “long-term residency in Chongqing” was as follows: residence within a 30-minute travel time from the delivery hospital. A professional team consisting of research nurses with over 5 years of obstetric experience and GIS specialists conducted a two-person verification process to rigorously screen pregnant women residing in areas near the hospital. This was done by self-reported community affiliation from the pregnant women (they provided the name of their residential community to the research nurse through verbal statements, such as “I live in XX community.”), a two-person check of communities and commute time. This process aims to enhance geographical representativeness and reduce the risk of exposure misclassification caused by long-distance relocation. Complete medical records were obtained, including information on the adjusted covariates and outcomes related to placenta previa and placenta accreta without previa, to ensure a comprehensive analysis. The exclusion criteria were as follows: (i) women diagnosed with type 1 or type 2 diabetes mellitus before pregnancy; (ii) patients with chronic hypertension; (iii) women diagnosed with eclampsia. In the initial screening, a total of 3,894 pregnant women met the inclusion criteria. After applying the exclusion criteria, the final number of participants included in this study was 3,670. The study flowchart is shown in **Figure 1**.

### Data collection

All data for this study were sourced from the electronic medical records (EMR) database of the Women and Children’s Hospital of Chongqing Medical University. The data were collected by trained medical staff, who reviewed the medical records to extract basic information about pregnant women and pregnancy-related data. The List of abbreviations and medical records can be seen in **Supplementary Table S1** and **Supplementary Table S2**. The database was constructed by systematically compiling patient information from electronic records, ensuring comprehensive inclusion of detailed demographic and clinical characteristics. The clinical characteristics include age, BMI, primigravida, nulliparity, assisted reproductive technology, family history of hypertension, family history of hyperglycemia, scarred uterus, uterine fibroids, chorionic characteristics, and season of conception.

Our outcomes and variables were primarily derived from rigorous pathological or clinical diagnoses, which were subsequently verified through ICD code cross-checking. By “clinical assessment,” we refer to diagnoses made by a qualified obstetrician-gynecologist (ObGyn), based on a thorough evaluation, including medical history, clinical examination, and relevant diagnostic tests. Acknowledging the potential for discrepancies between ICD codes and clinical diagnoses, we implemented a robust cross-validation process. In this process, ICD codes were compared with the clinical diagnoses made by the ObGyn to ensure the accuracy and consistency of the data. Prior studies have shown that this dual-validation approach effectively minimizes diagnostic errors and enhances the overall validity of the outcome classification (13, 22). The diagnostic criteria for inclusion were carefully defined and consistently applied throughout the review process, ensuring that clinical data, especially the outcomes, were accurately ascertained.

### Outcomes

Placenta previa and placenta accreta are two distinct obstetric complications. However, both conditions involve abnormal placental implantation, and they often coexist. Moreover, there is an association between placenta accreta and the occurrence of placenta previa. In this study, we defined two outcomes: placenta previa (including cases coexisting with placenta accreta); and placenta accreta without placenta previa.

### Air pollution exposure assessment

Located in the southwestern part of China, Chongqing covers a total area of 82,400 square kilometers, approximately 75% of which is mountainous terrain. As of the end of 2021, the city is comprised of 38 districts and counties, with a total population reaching 31.2 million. Chongqing primarily consists of four regions: the central area, the western area, the northeastern area, and the southeastern area. The central area features relatively flat terrain, a more developed economy, and a higher degree of urbanization. In contrast, the other regions are predominantly mountainous, with slower economic development and a scarcity of medical resources.

**Figure 2:**
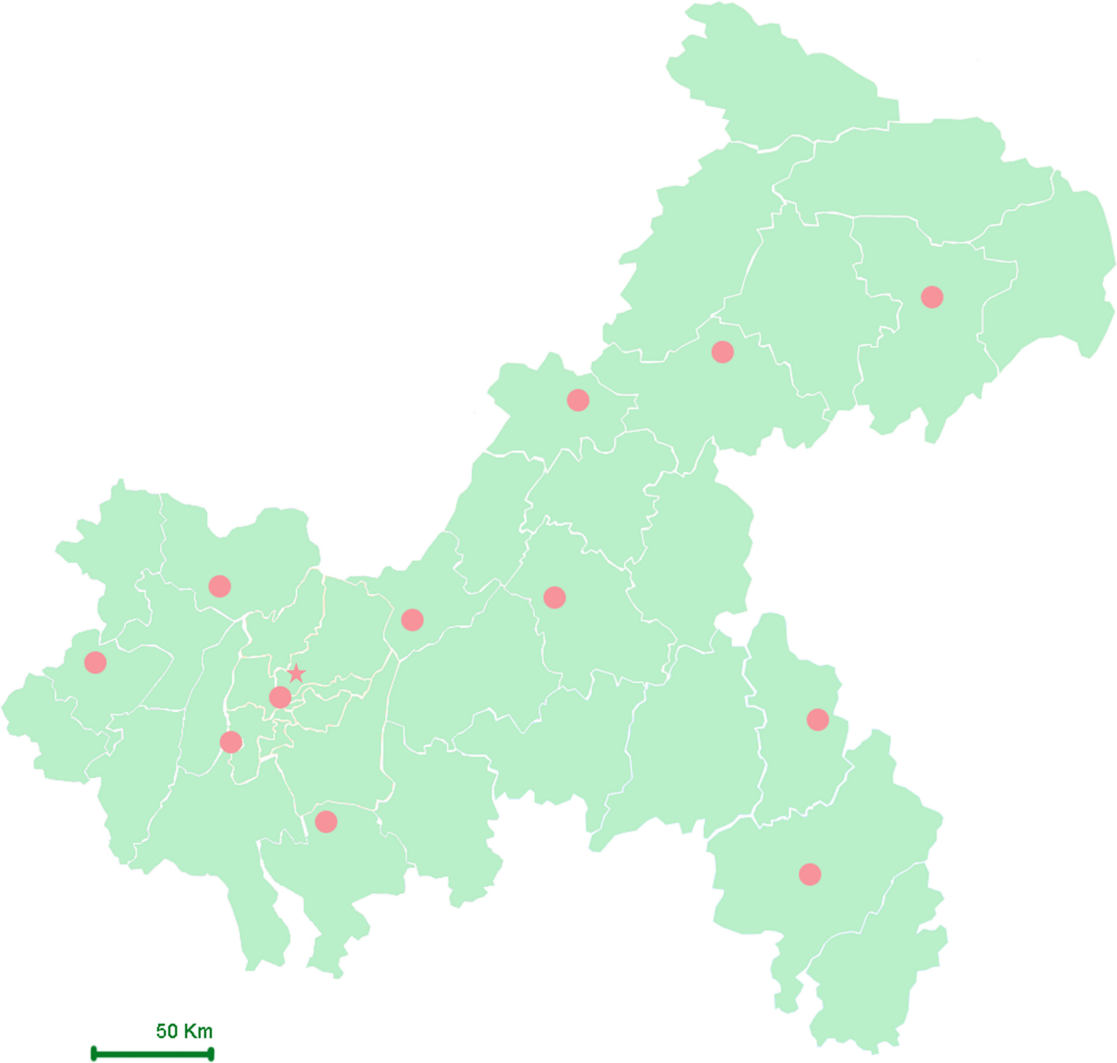
Geographical map and distribution of meteorological stations in Chongqing. Star (★): Represent the location of research hospital; Solid Circle (●): Represent the locations of meteorological stations.

Chongqing is equipped with 12 basic meteorological stations located in Fengjie, Wanzhou, Qianjiang, Youyang, Liangping, Changshou, Fengdu, Hechuan, Shapingba, Jiangjin, Qijiang, and Dazu (**Figure 2**). This study collected daily concentrations of ambient air pollutants for the period from December 2016 to December 2022 from the China Meteorological Data Sharing Service System, including particulate matter with an aerodynamic diameter ≤ 2.5μm or 10 μm (PM_2.5_ and PM_10_, respectively), nitrogen dioxide (NO_2_), sulfur dioxide (SO_2_), carbon monoxide (CO), and ozone (O_3_). Due to the need to protect patient privacy, we were unable to obtain the home addresses of each participant. Therefore, average exposure estimates were calculated using geocoding based on hospital locations, with exposure levels derived from data of the nearest monitoring stations, representing the pollution exposure of pregnant women who gave birth in those hospitals.

### Covariates

Covariates were pre-determined based on prior studies and included maternal age, pre-pregnancy body mass index (BMI, calculated as weight in kilograms divided by height in meters squared), primigravida status (first pregnancy) and nulliparity status (no previous childbirth), use of ART, family history of hypertension and hyperglycemia, chorionicity (dichorionic diamniotic [DCDA] vs. non-DCDA), presence of a scarred uterus, presence of uterine fibroids, conception season (spring, summer, autumn, or winter), and mean temperature (Tmean). Mean ambient temperature was included as a covariate because previous studies have suggested that temperature exposure may influence placental development and function, including the risk of abnormal implantation and placental complications (23–26). Multicollinearity among the covariates was assessed by calculating the Variance Inflation Factors (VIFs). VIFs for all variables were below the threshold of 10, suggesting that multicollinearity was not a concern in our model.

### Statistical analysis

In this study, descriptive statistics were presented as mean ± standard deviation or median and interquartile range (M (IQR)) for continuous variables, and as percentages for categorical variables. For variables that followed a normal distribution, one-way ANOVA was used for analysis; for non-normally distributed variables, the Kruskal-Wallis H test was applied. Categorical variables were assessed using the chi-square test. To evaluate the associations between ambient air pollutants and placenta previa and placenta accreta, exposure levels of air pollutants were analyzed both as continuous and categorical variables. First, multivariate logistic regression analyses were conducted to calculate ORs and corresponding 95% CIs to explore the associations between air pollutants as continuous variables and placenta previa and placenta accreta. We fitted each pollutant and its different exposure periods separately to facilitate comparisons of associations among pollutants and exposure periods. Placenta previa and placenta accreta, as well as complete placenta previa and marginal placenta previa, were used as dependent variables, while exposure levels of air pollutants were used as independent variables. Subgroup analyses were also performed in the absence of advanced maternal age, primigravida status, ART use, and uterine diseases. Additionally, to assess the exposure-response relationships, restricted cubic spline analysis was applied to evaluate the non-linear relationships between the six pollutants and the incidence of placenta accreta without previa during different periods, and to observe the trends of ORs. The reference level for ORs was set at the median pollution level, with the baseline at Y = 1. In further analyses, participants were divided into quartiles (Q1, Q2, Q3, Q4) based on individual concentrations of each pollutant during the 3 months preconception, the first trimester, and the second trimester, to examine the dose-response relationships between exposure to air pollutants and placenta previa and placenta accreta. Trend regression analysis was used, with the lowest quartile (Q1) as the reference group, to assess the trends in adverse pregnancy outcomes from Q1 to Q4. ORs and 95% CIs were estimated. The study also adjusted for maternal characteristics related to the risks of placenta previa and placenta accreta. When analyzing exposure during the second trimester, women with preterm birth (<29 weeks of gestation) were excluded. To assess the robustness of the results, a dual-pollutant model was constructed to evaluate the independent associations between air pollutants and placenta previa and placenta accreta. Pollutants with correlation coefficients higher than 0.9 were not fitted simultaneously. All p-values were two-sided, with *p* < 0.05 considered statistically significant. All analyses were conducted using SPSS 26.0 and R 4.4.2.

## Results

In this study, a total of 3,670 pregnant women with twin pregnancies were included, of whom 119 were diagnosed with placenta previa and 534 with placenta accreta without previa. Baseline characteristics are shown in **Table 1**. Among those with placenta previa, 51 cases were complete placenta previa, 64 cases were marginal placenta previa, and the remainder were other types of placenta previa (**Supplementary Table S3**).

**Table 1:** Population characteristics of placental diseases in twin pregnancies.

Characteristics	All participants (n= 3,670)	Participants without placenta previa and accreta (n= 3,017)	Participants with placenta previa (n= 119)	Participants with placenta accreta without previa (n= 534)
Age, M (Q_1_, Q_3_)	31.00 [28.00,33.00]	31.00 [28.00,33.00]	31.00 [29.00,34.00]	32.00 [29.00,34.00]
BMI, M (Q_1_, Q_3_)	21.26 [19.56,23.33]	21.23 [19.53,23.23]	21.48 [19.53,23.73]	21.55 [19.76,23.83]
Primigravida, n (%)	1723 (46.95)	1465 (48.56)	59 (49.58)	199 (37.27)
Nulliparity, n (%)	3009 (81.99)	2482 (82.27)	96 (80.67)	431 (80.71)
ART, n (%)	2598 (70.79)	2075 (68.78)	95 (79.83)	428 (80.15)
Family history of hypertension, n (%)	487 (13.27)	389 (12.89)	19 (15.97)	79 (14.79)
Family history of hyperglycemia, n (%)	231 (6.29)	184 (6.10)	4 (3.36)	43 (8.05)
Scarred uterus, n (%)	154 (4.20)	117 (3.88)	8 (6.72)	29 (5.43)
Uterine fibroids, n (%)	340 (9.26)	263 (8.72)	15 (12.61)	62 (11.61)
Chorionic, n (%)				
DCDA	2986 (81.36)	2436 (80.74)	96 (80.67)	454 (85.02)
Non-DCDA	684 (18.64)	581 (19.26)	23 (19.33)	80 (14.98)
Season of conception (n, %)				
Spring (March–May)	953 (25.97)	790 (26.19)	32 (26.89)	138 (25.84)
Summer (June–August)	920 (25.07)	761 (25.22)	27 (22.69)	135 (25.28)
Fall (September–November)	1042 (28.39)	853 (28.27)	33 (27.73)	152 (28.46)
Winter (December–February)	755 (20.57)	613 (20.32)	27 (22.69)	109 (20.41)

BMI, Body mass index; ART, Assisted Reproductive Technology; DCDA, Dichorionic-Diamniotic.

**Table 2** summarizes the distribution of six air pollutants across different time periods. During the 3 months preconception, the average concentrations of the pollutants were as follows: PM_2.5_ 43.53 μg/m³, PM_10_ 68.48 μg/m³, SO_2_ 13.16 μg/m³, NO_2_ 33.00 μg/m³, CO 1.29 mg/m³, and O_3_ 15.35 μg/m³. In the first trimester, the average concentrations were: PM_2.5_ 43.52 μg/m³, PM_10_ 68.49 μg/m³, SO_2_ 13.14 μg/m³, NO_2_ 33.02 μg/m³, CO 1.29 mg/m³, and O_3_ 15.27 μg/m³. In the second trimester, the average concentrations were: PM_2.5_ 42.15 μg/m³, PM_10_ 66.57 μg/m³, SO_2_ 12.84 μg/m³, NO_2_ 32.46 μg/m³, CO 1.28 mg/m³, and O_3_ 15.75 μg/m³. Throughout the entire exposure period of the study, PM_2.5_ and PM_10_ exhibited a very high Pearson correlation (r > 0.90). Additionally, PM_2.5_ and PM_10_ showed significant correlations with SO_2_ and NO_2_. Meanwhile, CO demonstrated progressively stronger correlations with other pollutants (except O_3_) over time, a trend that continued from the 3 months preconception through the second trimester. In stark contrast, O_3_ showed a clear negative correlation with other pollutants, particularly with NO_2_. Specifically, the Pearson correlation coefficients between O_3_ and NO_2_ were -0.851, -0.666, -0.771, and -0.824 during the first 20 weeks of pregnancy, the 3 months preconception, the first trimester, and the second trimester, respectively (see **Supplementary Table S4**).

**Table 2:** Exposure to ambient air pollution distribution.

Air pollutant species	Minimum	25th %tile	Median	75th %tile	Maximum	Mean	SD
3 months preconception							
PM_2.5_	26.82	36.48	39.69	49.80	70.49	43.53	9.29
PM_10_	43.54	58.80	63.82	76.74	103.52	68.48	12.63
SO_2_	9.23	11.86	13.01	14.44	17.89	13.16	1.81
NO_2_	28.32	31.29	33.01	34.70	39.33	33.01	2.38
CO	1.18	1.24	1.28	1.32	1.44	1.29	0.06
O_3_	11.96	14.56	15.32	16.18	17.92	15.35	1.16
Trimester 1							
PM_2.5_	26.02	36.01	39.05	52.15	70.49	43.52	10.45
PM_10_	42.88	58.17	62.95	79.54	103.52	68.49	14.15
SO_2_	9.26	11.69	12.88	14.62	17.89	13.14	1.97
NO_2_	26.24	31.29	33.03	34.87	39.18	33.02	2.65
CO	1.16	1.24	1.29	1.33	1.44	1.29	0.07
O_3_	11.96	14.30	15.13	16.26	19.91	15.27	1.38
Trimester 2							
PM_2.5_	25.77	35.01	38.43	50.01	70.24	42.15	10.46
PM_10_	42.88	57.10	61.83	76.92	103.38	66.57	14.08
SO_2_	9.23	11.20	12.49	14.45	17.86	12.84	2.02
NO_2_	25.24	30.54	32.49	34.74	39.33	32.46	2.88
CO	1.15	1.22	1.27	1.33	1.44	1.28	0.07
O_3_	11.96	14.62	15.39	16.62	21.29	15.75	1.69

PM_2.5_, fine particulate matter; PM_10_, inhalable particulate matter; SO_2_, sulfur dioxide; NO_2_, nitrogen dioxide; CO, carbon monoxide; O_3_, ozone.

**Table 3** presents the results of the association analysis between exposure to six pollutants during pregnancy and the risk of placenta previa and placenta accreta without previa, assessed using multivariate logistic regression. The results showed no significant associations between exposure levels of the six pollutants and the risk of placenta previa during the 3 months preconception, the first trimester, or the second trimester. Further subtype analysis focused on the two main types of placenta previa: marginal placenta previa and complete placenta previa. For marginal placenta previa, exposure to NO_2_ during the 3 months preconception was found to be protective, with an OR of 0.869 (95% CI: 0.769–0.979); no significant associations were found for the other pollutants or exposure periods. For complete placenta previa, no significant associations were observed between the six pollutants and risk across all exposure periods (see **Supplementary Table S6**).

**Table 3:** Ambient air pollution exposure and risk of placenta previa and placenta accreta in twin pregnancy by window of exposure.

Air pollutants	Placenta previa			Placenta accreta without previa		
	3 months preconception	Trimester 1	Trimester 2	3 months preconception	Trimester 1	Trimester 2
	aOR (95% CI)	aOR (95% CI)	aOR (95% CI)	aOR (95% CI)	aOR (95% CI)	aOR (95% CI)
PM2.5	0.997 (0.975,1.018)	1.007 (0.988,1.027)	0.997 (0.975,1.019)	0.979 (0.968,0.990)	0.992 (0.983,1.002)	0.997 (0.988,1.007)
PM10	0.999 (0.983,1.014)	1.005 (0.991,1.019)	0.999 (0.983,1.015)	0.986 (0.978,0.994)	0.995 (0.988,1.002)	0.999 (0.992,1.006)
SO2	0.989 (0.886,1.104)	1.041 (0.941,1.155)	0.999 (0.892,1.117)	0.892 (0.843,0.943)	0.970 (0.922,1.022)	1.018 (0.971,1.068)
NO2	0.983 (0.900,1.072)	1.028 (0.953,1.112)	0.994 (0.908,1.086)	0.913 (0.873,0.954)	0.978 (0.941,1.017)	1.020 (0.987,1.055)
CO	1.297 (0.031,51.232)	1.835 (0.074,48.329)	1.300 (0.027,57.308)	0.085 (0.013,0.560)	0.935 (0.177,4.986)	3.139 (0.724,13.746)
O3	1.035 (0.871,1.236)	0.952 (0.824,1.094)	1.129 (0.742,1.732)	1.141 (1.043,1.250)	1.082 (1.008,1.160)	0.969 (0.914,1.026)

PM_2.5_, fine particulate matter; PM_10_, inhalable particulate matter; SO_2_, sulfur dioxide; NO_2_, nitrogen dioxide; CO, carbon monoxide; O_3_, ozone.

The adjusted odds ratios (aOR) were calculated after controlling for maternal age, pre-pregnancy body mass index (BMI), primigravida and nulliparity status, use of assisted reproductive technology (ART), family history of hypertension, family history of hyperglycemia, presence of a scarred uterus and uterine fibroids, chorionicity, conception season, and mean temperature (Tmean).

In the analysis of placenta accreta without previa, exposure to pollutants such as PM_2.5_, PM_10_, SO_2_, NO_2_, and CO during the 3 months preconception appeared to act as protective factors. Specifically, the aORs were: PM_2.5_ (aOR 0.979, 95% CI: 0.968–0.990), PM10 (aOR 0.986, 95% CI: 0.978–0.994), SO2 (aOR 0.892, 95% CI: 0.843–0.943), NO2 (aOR 0.913, 95% CI: 0.873–0.954), and CO (aOR 0.085, 95% CI: 0.013–0.560). In contrast, exposure to O_3_ during the 3 months preconception and the first trimester significantly increased the risk of placenta accreta without previa, with aORs of 1.141 (95% CI: 1.043–1.250) and 1.082 (95% CI: 1.008–1.160), respectively. However, no significant associations were found between the six pollutants and placenta accreta without previa during the second trimester.

**Figure 3:**
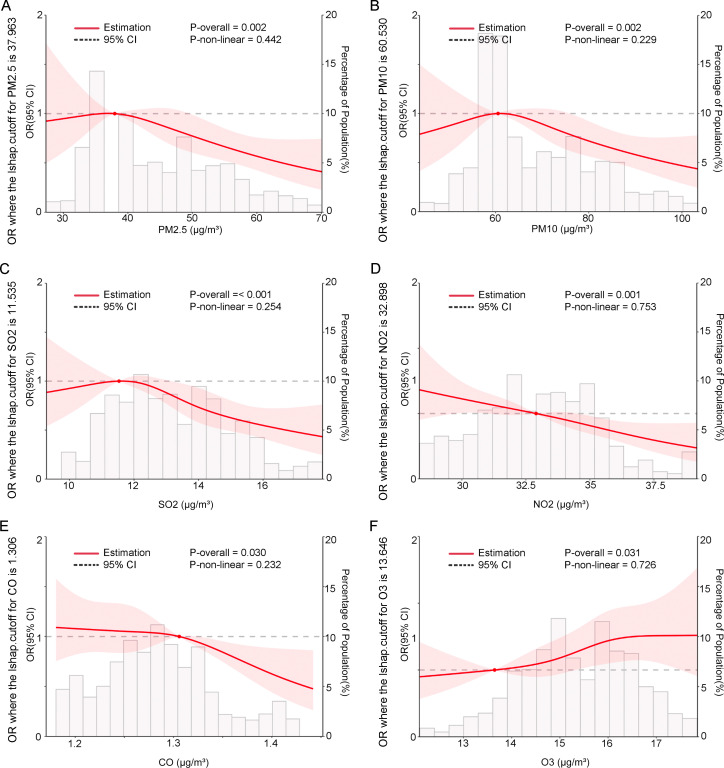
In twin pregnancies, the associations between six pollutants during the 3 months before conception and the odds ratios (OR) of placenta accreta without previa were modeled using restricted cubic splines. The pollutants included **(A)** PM_2.5_, **(B)** PM_10_, **(C) **SO_2_, **(D)** NO_2_, **(E)** CO_,_ and **(F) **O_3_. The reference level for OR was set at the median pollution concentration. The baseline was set at Y = 1. These curves were adjusted for maternal age, pre-pregnancy body mass index (BMI, calculated as weight in kilograms divided by height in meters squared), primigravida and nulliparity status, use of assisted reproductive technology (ART), family history (hypertension, hyperglycemia), presence of a scarred uterus and uterine fibroids, chorionicity (dichorionic diamniotic [DCDA], non-DCDA), conception season (spring, summer, autumn, or winter), and mean temperature (Tmean) . P-overall tests the overall significance of the relationship (linear and nonlinear); P-nonlinear tests whether the relationship is nonlinear. The shaded area represents the 95% confidence interval.

To further visualize the impact of the six pollutants on placenta accreta without previa across different periods, RCS analysis was conducted. Results showed significant associations between exposure to all six pollutants and the risk of placenta accreta without previa during the 3 months preconception (*p* for all < 0.05). However, no nonlinear relationships were observed between the pollutants and risk (*p* for nonlinearity > 0.05), although the 95% CIs were wide (**Figure 3**). RCS plots for other periods are shown in **Supplementary Figures S1**-**S2**.

For trend regression analysis, pollution data were categorized into four groups (**Supplementary Table S5**). For placenta previa, after adjusting for confounding factors, no significant associations were found between the six pollutants and placenta previa across all periods. However, during the second trimester, PM_2.5_ and PM_10_ were protective factors in the third quartile subgroup, with ORs of 0.464 (95% CI: 0.262–0.824) and 0.494 (95% CI: 0.281–0.869), respectively. In contrast, O_3_ in the second quartile subgroup was a risk factor for placenta previa, with an OR of 2.237 (95% CI: 1.315–3.804). During the 3 months before conception, exposure to PM_2.5_, PM_10_, SO_2_, and NO_2_ was significantly associated with a reduced risk of placenta accreta without previa, with all four pollutants showing protective associations (ORs ranging from 0.84 to 0.86). Although the overall association between CO and placenta accreta without previa was not significant during the 3 months before conception, a protective association was still observed in the highest exposure group (Q4), with an OR of 0.711. In contrast, O_3_ remained a significant risk factor during both the 3 months preconception and the first trimester. Notably, during the second trimester, NO_2_ emerged as a risk factor for placenta accreta without previa, with an OR of 1.092 (95% CI: 1.000–1.192) (**Supplementary Table S6**). Additionally, the associations between complete placenta previa, marginal placenta previa, and the six categorized pollutants across different periods are shown in **Supplementary Tables S7**, **8**.

Through stratified analyses, this study further explored the differential impacts of the six pollutants on placenta previa and placenta accreta without previa across different periods, specifically among pregnant women without advanced maternal age, primigravidae, those without ART use, and those without a history of uterine diseases (no scarred uterus or uterine fibroids). Among non-elderly pregnant women, none of the six pollutants showed significant associations with placenta previa across any period. However, during the 3 months preconception, exposure to PM_2.5_, PM_10_, SO_2_, NO_2_, and CO were identified as risk factors for placenta accreta without previa after adjusting for confounding factors. For nulliparity, among the six pollutants, only exposure to NO_2_ during the second trimester was associated with an increased risk of placenta previa, while no significant associations were observed for other pollutants or periods. Additionally, none of the six pollutants showed significant associations with placenta accreta without previa across any period. In twin pregnancies without ART use, none of the six pollutants showed significant associations with placenta previa across any period. However, during the first trimester, exposure to PM_2.5_ and PM_10_ were identified as risk factors for placenta accreta without previa after adjustment, with aORs of 1.023 (95% CI: 1.002–1.045) and 1.018 (95% CI: 1.002–1.034), respectively. Lastly, among women without a history of uterine diseases, none of the six pollutants showed significant associations with placenta previa across any period. However, most pollutants exhibited protective associations against placenta accreta without previa across various periods (see **Supplementary Tables S8**-**S11**). These findings suggest that the impacts of air pollutants on placenta previa and placenta accreta without previa vary across different populations and exposure periods, indicating that specific subgroups and exposure windows may be more susceptible to pollutant associations.

Finally, the robustness of pollutant associations was assessed through dual-pollutant models (see **Supplementary Tables S12**-**S14**). For placenta previa, none of the six pollutants showed significant associations across any period after adjustment. For placenta accreta without previa, during the 3 months preconception, PM_2.5_, PM_10_, SO_2_, and NO_2_ shifted from being protective to non-significant after adjusting for other pollutants, although they generally remained protective in most cases. CO was no longer associated with placenta accreta without previa after adjustment during this period. O_3_ remained a risk factor for placenta accreta without previa during the 3 months preconception and the first trimester in most cases, although it was non-significant in some scenarios after adjusting for other pollutants. Notably, during the second trimester, after adjusting for PM_2.5_ and PM_10_, both NO_2_ and CO emerged as risk factors for placenta accreta without previa.

## Discussion

Our study aimed to explore the relationship between air pollution and placental diseases, with a focus on twin pregnancies. We found that twin pregnancies were not significantly associated with placenta previa risk during the 3 months preconception, second trimester, or the overall early pregnancy period. This result is consistent with studies on singleton pregnancies, which also found no strong associations between air pollution exposure during early pregnancy and the risk of placenta previa (20, 27). However, our study expands on existing literature by focusing specifically on twin pregnancies, which have distinct placental and vascular characteristics that may influence their susceptibility to environmental exposures. This highlights the importance of considering pregnancy type in studies of air pollution and placental diseases. In terms of comparable evidence, a study conducted in Japan on singleton pregnancies found a positive correlation between exposure to particulate matter and ozone during early pregnancy (gestational weeks 0-4) and the risk of placenta previa. Additionally, exposure to NO_2_ and SO_2_ was associated with an increased risk, though no associations were found with exposure during later stages of early pregnancy or the second trimester (20). Another study, which used proximity to major roads as a proxy for air pollution, found no link between this exposure and placenta previa risk (27). While these studies provide valuable insights into the relationship between air pollution and placenta previa, they primarily focus on singleton pregnancies, and their findings may not be directly applicable to twin pregnancies. Our study, while not further classifying early pregnancy exposure, provides important contributions by extending the research to twin pregnancies. Despite the limitations of not precisely assessing the timing of exposure, our findings underscore the need for further research into the impact of environmental factors on twin pregnancies, which may differ biologically and physiologically from singleton pregnancies.

The finding that exposure to PM_2.5_, PM_10_, SO_2_, and NO_2_ during the 3 months preconception period may act as protective factors against placenta accreta in twin pregnancies is intriguing, as it contradicts the prevailing view that air pollution generally exerts harmful effects on health. Several explanations may underlie these results, but they should be interpreted with caution due to potential statistical artifacts, particularly the limitations of exposure assessment. The study’s reliance on group-level air pollution data, sourced from fixed monitoring stations near the delivery hospital, introduces the risk of non-differential exposure misclassification, where individuals may be inaccurately assigned exposure levels based on hospital referral areas rather than their actual residential locations. To minimize exposure misclassification and enhance geographical representativeness, our study formed a team of experienced obstetric nurses and GIS specialists, who conducted a two-person verification process to rigorously screen participants. The inclusion criteria required that the pregnant women’s residence be within a 30-minute commute to the delivery hospital. This standard was determined based on self-reported community affiliation (by reporting the name of residential community). The core goal of this screening process was to reduce exposure misclassification caused by long-distance relocations or living far from monitoring stations. Epidemiological studies have shown that pregnant women rarely relocate, and most relocations are short-distance moves, with the majority occurring within the same community or within a 3 km radius (28, 29). Evidence also indicates that when relocation distances are short, the error introduced by using monitoring data near the delivery address is relatively small (30). For example, a study in England showed that when the relocation distance is less than 5 km, the difference in PM_10_ exposure levels is typically less than 5% (31). Therefore, non-differential exposure misclassification is a reasonable assumption in the context of this study. This misclassification is more likely to dilute associations, potentially underestimating the true effect of air pollution on placental diseases, future studies should aim to incorporate more precise individual-level exposure data to minimize this limitation. This is compounded by the retrospective design, which increases the likelihood of unmeasured confounders, further complicating the attribution of causality. As such, while the observed protective effect is thought-provoking, the possibility of a false positive cannot be ruled out, and further investigation is warranted (32). From a biological standpoint, the protective effect of PM_2.5_ and PM_10_ could be related to their influence on the endometrial microenvironment, which plays a critical role in placental implantation. In twin pregnancies, where placental development is inherently more complex due to the shared uterine environment, pollutants like PM_2.5_ and PM_10_ may exert indirect effects by influencing angiogenesis and cell proliferation within the endometrial lining (33). These processes are essential for establishing optimal receptivity for placental accreta, which may be enhanced by the pollutants’ modulation of vascular function. Specifically, PM2.5 and PM10, which are fine particulate matter, are known to penetrate deep into the respiratory tract and may influence systemic inflammation, which could indirectly affect placental function by promoting angiogenesis (34–36). While this mechanism is plausible, it remains speculative and needs further empirical validation (37). Similarly, SO_2_ and NO_2_, pollutants primarily associated with traffic and industrial emissions, could affect the immune microenvironment during the preconception period. SO_2_ is known to influence vascular health and endothelial function (38), which could affect placental accreta by modulating blood flow and angiogenesis within the endometrium. Exposure to SO_2_ may lead to subtle changes in immune cell activity and cytokine production, which could alter the maternal immune response, promoting an environment conducive to placental attachment (39). Similarly, NO_2_ has been linked to alterations in immune modulation, with potential impacts on the immune tolerance mechanisms necessary for successful placentation. By modulating the inflammatory response, NO_2_ may alter the immune environment at the maternal-fetal interface, potentially facilitating normal placental accreta, particularly in the context of twin pregnancies where trophoblast invasion and placental formation are more complex (40). However, it is important to note that these biological mechanisms are speculative and require further research. While there is substantial evidence that air pollution affects immune function, angiogenesis, and vascular health, the specific impacts on placental diseases such as accreta remain unclear. Further studies are needed to elucidate the direct biological pathways by which these pollutants may influence placental development. In contrast, O_3_ exposure consistently emerged as a significant risk factor for placenta accreta, a finding that aligns with existing research. O_3_ induces oxidative stress and inflammation, which can disrupt placental function and contribute to placental abnormalities (21, 41). This consistent risk is in line with the growing body of evidence suggesting that ozone, unlike the other pollutants in this study, exerts harmful effects on placental health.

For the second trimester, the single-pollutant model, consistent with Michikawa’s study (20), found no significant associations between the six pollutants and placenta accreta. However, the dual-pollutant model further revealed that NO_2_ and CO became risk factors for placenta accreta after adjusting for PM_2.5_ and PM_10_ during the second trimester (18). This could be due to the impact of these pollutants on vascular function and blood flow regulation. Both NO_2_ and CO are known to induce vascular constriction and endothelial dysfunction, leading to reduced uterine blood flow (38, 42). Insufficient blood supply to the endometrium may impair proper placental implantation, causing the placenta to implant abnormally in the lower uterine segment or invade too deeply into the uterine wall, characteristic of placenta accreta. Additionally, CO can cause hypoxia by reducing oxygen delivery to tissues, further disrupting trophoblast invasion and placental development (43–45). These combined effects may explain the observed increased risk of placenta accreta. For placenta accreta without previa, we hypothesize that systemic inflammation induced by air pollutants affects the endometrium (46, 47), leading to poor decidualization (48). An experimental study in rats reported an association between preconception and pregnancy exposure to PM_2.5_ and placental inflammatory responses (49); thus, it is reasonable to hypothesize that inflammation induced by certain pollutants also occurs in the endometrium. Further experimental and epidemiological studies are needed to determine whether exposure to O_3_ and other air pollutants is associated with placenta accreta.

The strengths of this study lie in the use of a high-quality clinical database, which includes only cases of placenta previa and placenta accreta diagnosed by obstetric specialists. Additionally, the database covers information on potential risk factors for placenta previa and placenta accreta, such as maternal age, primigravida status, history of ART use, and uterine diseases. Therefore, we were able to carefully assess whether the positive associations between air pollutant exposure and placenta previa or placenta accreta were explained by these confounding factors through stratified analyses. Initially, we considered maternal age and parity, as advanced maternal age and multiparity are potential risk factors for placenta previa and placenta accreta. Specifically, analyses restricted to nulliparity were not only aimed at preventing confounding by parity but also at limiting participants to those without a history of cesarean section or placenta previa or accreta, as we lacked information on these risk factors. However, after adjusting for these factors, we found that among women aged <35 years and nulliparity, for placenta previa, the results remained insensitive except for NO_2_ in the second trimester, which emerged as a risk factor. For placenta accreta without previa, after restricting to women aged <35 years and nulliparity, the risks associated with the six pollutants changed. Specifically, PM_2.5_, PM_10_, SO_2_, NO_2_, and CO exposure during the 3 months preconception no longer showed significant protective effects in nulliparity with twin pregnancies and even shifted to risk factors in women aged <35 years with twin pregnancies. This may be because for women aged <35 years who are nulliparity their reproductive systems have not undergone the physiological adjustments of multiple pregnancies, and the adaptability and protective mechanisms of the placenta may be relatively weaker. Additionally, the endometrium and placenta in primigravidae may be more sensitive to environmental pollutants (50, 51). Exposure to these pollutants during the 3 months preconception may interfere with normal placental development by inducing oxidative stress and inflammatory responses. In twin pregnancies, where the placental burden is heavier, pollutant exposure may further exacerbate placental inflammatory responses, leading to placental dysfunction. Therefore, in these two subgroups of women with twin pregnancies, preconception exposure to these pollutants may weaken the protective mechanisms of the placenta and even shift to risk factors, increasing the risk of placenta accreta.

Additionally, previous studies have suspected that the history of infertility treatment with ART and uterine diseases may confound the occurrence of placental diseases (20, 52–54). Therefore, this study further conducted stratified analyses to explore the associations between the six pollutants and placenta previa in twin pregnancies without a history of ART or uterine diseases. The results showed no significant associations, consistent with previous sensitivity analyses in singleton pregnancies (20). For placenta accreta without previa, the impacts of the six pollutants showed opposite changes after restricting the analyses to pregnancies without a history of ART or uterine diseases. Specifically, in the first trimester, PM_2.5_ and PM_10_ were risk factors for placenta accreta without previa among women without a history of ART. This may be because ART itself may reduce the risk of abnormal placental accreta by optimizing embryo selection and the uterine environment. Women without ART history may lack this protective mechanism, making the placenta more susceptible to the effects of pollutant-induced oxidative stress and inflammatory responses during early development, thereby increasing the risk of abnormal placental invasion into the myometrium. In contrast, for women without a history of uterine diseases, most pollutants showed protective effects against placenta accreta without previa across various periods. This may be because uterine diseases can alter the endometrial environment, increasing the risk of abnormal placental implantation. In women without uterine diseases, the endometrial environment is relatively healthy, providing a better foundation for placental development. Therefore, when exposed to pollutants, the placenta may adapt to environmental stress through its own regulatory mechanisms, and may even induce compensatory enhancements in placental angiogenesis and function to reduce the risk of abnormal implantation. Additionally, previous studies have often been limited to early pregnancy windows, but pollutant exposure may continue to affect angiogenesis or immune regulation, exacerbating implantation-related pathological risks (55). The biological effects of such exposure are cumulative, with sustained exposure during mid-pregnancy potentially leading to the additive effects of inflammation or oxidative damage, which can disrupt normal placental function, especially in twin pregnancies (55, 56). In contrast, this study extends the exposure assessment to the preconception and mid-pregnancy periods, which is more likely to capture cumulative risks and provide a more comprehensive perspective on the pathological mechanisms.

### Limitations

However, this study also has several limitations. First, data were sourced from fixed monitoring stations near the delivery hospital, with air pollution estimates averaged over hospital referral areas. Due to the lack of individual residential addresses, exposure misclassification may have occurred, which could affect the validity of the study results, as we could not directly measure individual exposure levels. This also limits our ability to conduct sensitivity analyses based on residential location. Although integrating global grid models allows for comparative studies, the lack of individual residential coordinates prevents us from constructing a residential exposure reference framework and makes it difficult to distinguish between model differences and residential misclassification effects. Moving forward, future studies should aim to collect more detailed residential data, including potentially geocoded addresses or neighborhood centroids where ethically permissible. Further efforts should be made to integrate high-quality global and China-specific environmental models for comparative studies, such as high-resolution spatiotemporal models for PM2.5, ozone, and temperature. This would enable a more accurate exposure assessment and the possibility of performing sensitivity analyses using spatial pollutant grids, improving the robustness and generalizability of the findings. Secondly, this study is limited by its retrospective design and ethical review requirements, which prevented the systematic collection of data on changes in the residential addresses of pregnant women during their pregnancy (such as the number of relocations, timing, and distance). Although an attempt was made to reduce the risk of long-distance relocations using a 30-minute travel circle screening strategy, the actual relocation rate and directionality changes could not be quantified. Additionally, due to the use of a single institution-based delivery cohort design, the study did not assess institutional transfer behavior of pregnant women from their first antenatal care (ANC) visit to delivery. These factors could affect the continuity of exposure accumulation during pregnancy. Although existing studies suggest a low relocation rate, future research should consider more detailed data collection and spatial analysis methods to improve the accuracy and continuity of exposure assessment. The study did not account for indoor air quality, which may differ significantly from outdoor pollution and could influence the associations between air pollutants and placental diseases. The absence of this data may limit the accuracy of our findings. Additionally, important variables such as smoking during pregnancy, prior uterine surgeries, and socio-economic status (SES) were not included. The lack of SES data, which is often unavailable in medical records, may contribute to residual confounding. These unmeasured factors may limit our ability to fully assess the associations. Unmeasured factors like area-level socioeconomic status, the mother’s occupation, activity patterns, and time spent indoors versus outdoors also affect exposure levels. For instance, outdoor workers may experience higher exposure to pollutants, and residential mobility, where women relocate to reduce exposure, could lead to discrepancies between estimated and actual exposure. These omissions and unaccounted-for factors may contribute to potential misclassification of environmental exposures and limit the generalizability of the study’s findings. Second, the lack of specific residential addresses prevented the use of more precise exposure estimation methods like inverse distance weighting (IDW). While some studies suggest IDW estimates and nearby meteorological station data are comparable, biases such as clustering effects still need consideration (57). Future research should integrate methods like IDW combined with random forest interpolation and address factors like residential mobility and clustering around hospitals. These refinements would improve exposure accuracy, enhancing the precision of models and providing a stronger basis for understanding the health risks of air pollution, ultimately guiding better intervention strategies for adverse pregnancy outcomes. Third, although we considered several potential confounding factors, some important information was not collected, such as the impact of smoking during pregnancy and a history of uterine surgery, both of which are potential risk factors for placenta previa and placenta accreta (20). Another limitation is that our database did not allow us to link information between a participant’s first pregnancy and subsequent pregnancies. However, despite not accounting for the internal correlation of re-entering participants, our results explored the impact of air pollutants on twin pregnancies stratified by primigravida status, ART use, and absence of uterine diseases. Additionally, air pollution levels in Chongqing are relatively high compared to those in Western countries, which may overestimate or underestimate the impact on placenta previa and placenta accreta in twin pregnancies. The characteristics of our study participants may differ from the general pregnant population due to the nature of the collaborating hospitals. Therefore, we should be cautious about the generalizability of the results. Lastly, while the study is based on robust observational data, it cannot establish causality. As a retrospective design, it does not account for potential variations in air pollution exposure over time or capture the effects of cumulative exposure. Future research could benefit from exposure lag analysis, such as Distributed Lag Non-Linear Models (DLNM), to better assess delayed and cumulative effects. Longitudinal or experimental studies are needed to confirm the mechanisms suggested by these results and provide stronger evidence of causality.

## Data Availability

The raw data supporting the conclusions of this article will be made available by the authors, without undue reservation.
