# Supplementary material for: The differential impacts of environmental air pollution exposure on the risk of placenta previa and placenta accreta in twin pregnancies
Source: Front Endocrinol (Lausanne). 2025 Sep 8;16:1624480. doi: 10.3389/fendo.2025.1624480 (PMC12450698; doi:10.3389/fendo.2025.1624480)
Supplement: Supplementary file 1 [file DataSheet1.docx]

**The differential impacts of environmental air pollution exposure on the risk of placenta previa and placenta accreta in twin pregnancies**

Wei-Zhen Tang ^a,b,^**^¶^**, Hong Chen ^c,d,^**^¶^**, Hong-Yu Xu ^a,b^, Qin-Yu Cai ^a,d^, Niya Zhou ^a,b^, Yi-Fan Zhao ^c^, Bo-Yuan Deng ^c^, Xu Zhang ^c^, Fei Han ^d^, Tai-Hang Liu^b,c,^*, Zhen Chen ^a,b,^*

**Affiliations:**

^a^ Department of Obstetrics and Gynecology, Women and Children’s Hospital of Chongqing Medical University, Chongqing, 401147, China.

^b^ Chongqing Health Center for Women and Children, 401147, China.

^c^ School of Basic Medical Sciences, Chongqing Medical University, Chongqing, 400016, China.

^d^ The Joint International Research Laboratory of Reproduction and Development, Chongqing Medical University, Chongqing, 400016, China.

***Correspondence:**

*Tai-Hang Liu (E-mail Address: liuth@cqmu.edu.cn); Zhen Chen (E-mail Address: zhenzhenwyg@163.com). Box 197, Chongqing Medical University, No.1 Yixueyuan Rd, Chongqing, 400016, PR China. Tel.: +86 023 68485868.

**¶ W.Z. Tang and H. Chen contributed equally to this work.**

**Table S1** List of abbreviations

| **Abbreviation** | **Full Form** |
| --- | --- |
| ANOVA | Analysis of Variance |
| aOR | adjusted Odds Ratio |
| ART | Assisted Reproductive Technology |
| BMI | Body Mass Index |
| CI | Confidence Interval |
| CO | Carbon Monoxide |
| DCDA | Dichorionic Diamniotic |
| DLNM | Distributed Lag Non-Linear Models |
| EMR | Electronic Medical Records |
| HDP | Hypertensive Disorders of Pregnancy |
| ICD | International Classification of Diseases |
| IDW | Inverse Distance Weighting |
| IQR | Interquartile Range |
| IUGR | Intrauterine Growth Restriction |
| M | Median |
| NICE | National Institute for Health and Care Excellence |
| NICU | Neonatal Intensive Care Unit |
| NO₂ | Nitrogen Dioxide |
| O₃ | Ozone |
| OR | Odds Ratio |
| PM₁₀ | Particulate Matter ≤ 10 μm |
| PM₂.₅ | Particulate Matter ≤ 2.5 μm |
| RCOG | Royal College of Obstetricians and Gynaecologists |
| RCS | Restricted Cubic Spline |
| SD | Standard Deviation |
| SO₂ | Sulfur Dioxide |
| Tmean | Mean Temperature |

**Table S2.** The List of medical records

| **Full Form** |
| --- |
| Age |
| BMI |
| Primigravida |
| Nulliparity |
| Assisted reproductive technology |
| Family history of hypertension |
| Family history of hyperglycemia |
| Scarred uterus |
| Uterine fibroids |
| Chorionic |
| Season of conception |
| Placenta previa |
| Placenta accreta without previa |

**Table S3.** Characteristics of complete placenta previa and marginal placenta previa participants included in this study

| **Characteristics** | **Complete placenta previa**  **(n = 51)** | **Marginal placenta previa**  **(n = 64)** |
| --- | --- | --- |
| Age | 32.00[30.00,35.00] | 31.00 [29.00,33.00] |
| BMI, M (Q₁, Q₃) | 21.48[19.53,23.73] | 21.57 [19.41, 23.93] |
| Primigravida, n(%) | 22(43.14) | 36(56.25) |
| Nulliparity, n(%) | 42(82.35) | 51(79.69) |
| ART, n(%) | 43(84.31) | 49(76.56) |
| Family history of hypertension, n(%) | 5(9.80) | 13(20.31) |
| Family history of hyperglycemia, n(%) | 1(1.961) | 3(4.69) |
| Scarred uterus, n(%) | 4(7.84) | 10(15.63) |
| Uterine fibroids, n(%) | 5(9.80) | 2(3.13) |
| Chorionic, n(%) |  |  |
| DCDA | 42(82.35) | 51(79.69) |
| Non-DCDA | 9(17.65) | 13(20.31) |
| Season of conception(n, %) |  |  |
| Spring (March–May) | 13(25.49) | 19(29.69) |
| Summer (June–August) | 8(15.69) | 17(26.56) |
| Fall (September–November) | 19(37.26) | 13(20.31) |
| Winter(December–February) | 11(21.57) | 15(23.44) |

*p < 0.05

Abbreviation: BMI: Body mass index; ART: Assisted Reproductive Technology; DCDA: Dichorionic-Diamniotic

**Table S4** Spearman correlation coefficients among criteria pollutants during the 3 months preconception、Trimester 1and Trimester 2

| Air pollutant species | PM_2.5_ | PM_10_ | SO_2_ | NO_2_ | CO | O_3_ |
| --- | --- | --- | --- | --- | --- | --- |
| 3 months preconception |  |  |  |  |  |  |
| PM_2.5_ | 1 |  |  |  |  |  |
| PM_10_ | 0.996 | 1 |  |  |  |  |
| SO_2_ | 0.948 | 0.962 | 1 |  |  |  |
| NO_2_ | 0.892 | 0.900 | 0.942 | 1 |  |  |
| CO | 0.755 | 0.754 | 0.740 | 0.850 | 1 |  |
| O_3_ | -0.480 | -0.475 | -0.483 | -0.680 | -0.721 | 1 |
| Trimester 1 |  |  |  |  |  |  |
| PM_2.5_ | 1 |  |  |  |  |  |
| PM_10_ | 0.997 | 1 |  |  |  |  |
| SO_2_ | 0.958 | 0.968 | 1 |  |  |  |
| NO_2_ | 0.898 | 0.905 | 0.946 | 1 |  |  |
| CO | 0.827 | 0.831 | 0.825 | 0.866 | 1 |  |
| O_3_ | -0.573 | -0.574 | -0.595 | -0.771 | -0.732 | 1 |
| Trimester 2 |  |  |  |  |  |  |
| PM_2.5_ | 1 |  |  |  |  |  |
| PM_10_ | 0.997 | 1 |  |  |  |  |
| SO_2_ | 0.963 | 0.972 | 1 |  |  |  |
| NO_2_ | 0.898 | 0.900 | 0.942 | 1 |  |  |
| CO | 0.847 | 0.850 | 0.849 | 0.901 | 1 |  |
| O_3_ | -0.636 | -0.623 | -0.652 | -0.824 | -0.762 | 1 |

Abbreviation: PM_2.5_: fine particulate matter; PM_10_: inhalable particulate matter; SO_2_: sulfur dioxide; NO_2_: nitrogen dioxide; CO: carbon monoxide; O_3_: ozone

**Table S5.** Risk factor analysis of placenta previa and placenta accreta in twin pregnancies during the period of 3 months before conception, Trimester 1, and Trimester 2

| **EC** | **Placenta previa** | | | | **Placenta accreta without previa** | | | |
| --- | --- | --- | --- | --- | --- | --- | --- | --- |
|  | **Unadjusted Model** | | **Adjusted Model** | | **Unadjusted Model** | | **Adjusted Model** | |
|  | **OR (95% CI)** | ***p*-value** | **aOR (95% CI)** | ***p*-value** | **OR (95% CI)** | ***p*-value** | **aOR (95% CI)** | ***p*-value** |
| **PM_2.5_** |  |  |  |  |  |  |  |  |
| 3 months before conception |  |  |  |  |  |  |  |  |
| Q 1 | Reference |  | Reference |  | Reference |  | Reference |  |
| Q 2 | 1.044(0.621,1.754) | 0.871 | 1.020(0.602,1.729) | 0.942 | 0.879(0.685,1.126) | 0.307 | 0.833(0.645,1.075) | 0.160 |
| Q 3 | 1.144(0.689,1.901) | 0.603 | 1.167(0.700,1.947) | 0.554 | 0.753(0.584,0.972) | 0.029* | 0.720(0.556,0.934) | 0.013* |
| Q 4 | 0.935(0.549,1.593) | 0.805 | 0.904(0.513,1.594) | 0.728 | 0.649(0.499,0.845) | 0.001* | 0.586(0.441,0.778) | <0.001* |
| P for trend | 0.991(0.842,1.167) | 0.913 | 0.990(0.834,1.175) | 0.906 | 0.865(0.797,0.940) | 0.001* | 0.840(0.770,0.918) | <0.001* |
| Trimester 1 |  |  |  |  |  |  |  |  |
| Q 1 | Reference |  | Reference |  | Reference |  | Reference |  |
| Q 2 | 1.339(0.790,2.271) | 0.278 | 1.328(0.782,2.255) | 0.293 | 1.007(0.784,1.292) | 0.959 | 0.984(0.764,1.268) | 0.901 |
| Q 3 | 1.128(0.652,1.949) | 0.667 | 1.147(0.661,1.990) | 0.626 | 0.690(0.528,0.902) | 0.007* | 0.675(0.514,0.887) | 0.005* |
| Q 4 | 1.335(0.787,2.263) | 0.284 | 1.383(0.795,2.403) | 0.251 | 0.894(0.693,1.154) | 0.390 | 0.907(0.692,1.189) | 0.480 |
| P for trend | 1.069(0.907,1.259) | 0.426 | 1.084(0.912,1.289) | 0.360 | 0.931(0.858,1.011) | 0.090 | 0.933(0.855,1.018) | 0.119 |
| Trimester 2 |  |  |  |  |  |  |  |  |
| Q 1 | Reference |  | Reference |  | Reference |  | Reference |  |
| Q 2 | 0.667(0.398,1.118) | 0.124 | 0.656(0.387,1.112) | 0.118 | 0.957(0.742,1.236) | 0.737 | 0.949(0.729,1.235) | 0.695 |
| Q 3 | 0.474(0.268,0.840) | 0.010* | 0.464(0.262,0.824) | 0.009* | 0.768(0.589,1.002) | 0.052 | 0.765(0.584,1.002) | 0.052 |
| Q 4 | 0.869(0.536,1.408) | 0.568 | 0.841(0.505,1.402) | 0.507 | 0.964(0.747,1.244) | 0.777 | 0.930(0.709,1.220) | 0.602 |
| P for trend | 0.924(0.781,1.094) | 0.361 | 0.909(0.764,1.082) | 0.285 | 0.968(0.891,1.051) | 0.437 | 0.955(0.876,1.040) | 0.291 |
| **PM_10_** |  |  |  |  |  |  |  |  |
| 3 months before conception |  |  |  |  |  |  |  |  |
| Q 1 | Reference |  | Reference |  | Reference |  | Reference |  |
| Q 2 | 1.175(0.698,1.977) | 0.544 | 1.171(0.688,1.991) | 0.560 | 0.865(0.675,1.108) | 0.251 | 0.823(0.637,1.064) | 0.137 |
| Q 3 | 1.173(0.694,1.982) | 0.551 | 1.198(0.706,2.033) | 0.502 | 0.771(0.597,0.995) | 0.045* | 0.737(0.568,0.956) | 0.021* |
| Q 4 | 1.076(0.632,1.833) | 0.786 | 1.067(0.606,1.879) | 0.822 | 0.656(0.504,0.853) | 0.002* | 0.591(0.445,0.785) | <0.001* |
| P for trend | 1.021(0.867,1.202) | 0.806 | 1.022(0.861,1.212) | 0.807 | 0.871(0.802,0.946) | 0.001* | 0.846(0.775,0.924) | <0.001* |
| Trimester 1 |  |  |  |  |  |  |  |  |
| Q 1 | Reference |  | Reference |  | Reference |  | Reference |  |
| Q 2 | 1.253(0.741,2.120) | 0.400 | 1.224(0.722,2.076) | 0.452 | 1.020(0.794,1.310) | 0.877 | 0.998(0.773,1.287) | 0.986 |
| Q 3 | 1.089(0.633,1.872) | 0.758 | 1.099(0.637,1.896) | 0.734 | 0.731(0.560,0.953) | 0.021* | 0.718(0.548,0.941) | 0.016* |
| Q 4 | 1.297(0.769,2.186) | 0.330 | 1.337(0.775,2.307) | 0.296 | 0.900(0.696,1.162) | 0.418 | 0.914(0.698,1.197) | 0.513 |
| P for trend | 1.065(0.904,1.254) | 0.451 | 1.079(0.908,1.283) | 0.386 | 0.937(0.863,1.017) | 0.121 | 0.940(0.861,1.025) | 0.161 |
| Trimester 2 |  |  |  |  |  |  |  |  |
| Q 1 | Reference |  | Reference |  | Reference |  | Reference |  |
| Q 2 | 0.666(0.398,1.116) | 0.123 | 0.645(0.382,1.088) | 0.100 | 0.855(0.661,1.106) | 0.234 | 0.849(0.653,1.105) | 0.223 |
| Q 3 | 0.505(0.288,0.886) | 0.017* | 0.494(0.281,0.869) | 0.014* | 0.758(0.582,0.987) | 0.039 | 0.748(0.573,0.978) | 0.034 |
| Q 4 | 0.839(0.516,1.365) | 0.479 | 0.805(0.484,1.339) | 0.403 | 0.961(0.746,1.237) | 0.756 | 0.927(0.711,1.209) | 0.575 |
| P for trend | 0.918(0.775,1.087) | 0.319 | 0.903(0.759,1.075) | 0.251 | 0.976(0.899,1.060) | 0.567 | 0.963(0.884,1.049) | 0.391 |
| **SO_2_** |  |  |  |  |  |  |  |  |
| 3 months before conception |  |  |  |  |  |  |  |  |
| Q 1 | Reference |  | Reference |  | Reference |  | Reference |  |
| Q 2 | 1.015(0.604,1.705) | 0.955 | 0.960(0.561,1.644) | 0.883 | 0.865(0.675,1.107) | 0.249 | 0.796(0.614,1.031) | 0.084 |
| Q 3 | 1.218(0.738,2.010) | 0.441 | 1.231(0.743,2.040) | 0.421 | 0.743(0.576,0.959) | 0.023* | 0.710(0.547,0.922) | 0.010* |
| Q 4 | 0.876(0.509,1.507) | 0.632 | 0.828(0.465,1.474) | 0.521 | 0.649(0.498,0.845) | 0.001* | 0.583(0.439,0.776) | <0.001* |
| P for trend | 0.984(0.835,1.159) | 0.847 | 0.981(0.827,1.164) | 0.827 | 0.865(0.796,0.940) | 0.001* | 0.842(0.771,0.919) | <0.001* |
| Trimester 1 |  |  |  |  |  |  |  |  |
| Q 1 | Reference |  | Reference |  | Reference |  | Reference |  |
| Q 2 | 1.093(0.653,1.829) | 0.734 | 1.089(0.648,1.827) | 0.748 | 1.028(0.800,1.321) | 0.828 | 1.030(0.798,1.329) | 0.820 |
| Q 3 | 0.878(0.510,1.510) | 0.638 | 0.879(0.510,1.518) | 0.645 | 0.736(0.564,0.962) | 0.025* | 0.732(0.558,0.961) | 0.025* |
| Q 4 | 1.195(0.722,1.978) | 0.489 | 1.257(0.733,2.156) | 0.406 | 0.922(0.714,1.190) | 0.531 | 0.964(0.732,1.268) | 0.792 |
| P for trend | 1.036(0.880,1.219) | 0.671 | 1.046(0.880,1.244) | 0.606 | 0.945(0.870,1.025) | 0.173 | 0.951(0.872,1.038) | 0.261 |
| Trimester 2 |  |  |  |  |  |  |  |  |
| Q 1 | Reference |  | Reference |  | Reference |  | Reference |  |
| Q 2 | 1.186(0.716,1.964) | 0.507 | 1.166(0.703,1.936) | 0.552 | 1.174(0.904,1.523) | 0.229 | 1.149(0.882,1.497) | 0.304 |
| Q 3 | 0.659(0.367,1.184) | 0.163 | 0.656(0.364,1.180) | 0.159 | 0.974(0.744,1.276) | 0.851 | 0.953(0.725,1.252 | 0.729 |
| Q 4 | 1.044(0.621,1.755) | 0.870 | 1.018(0.598,1.734) | 0.948 | 1.197(0.923,1.552) | 0.176 | 1.162(0.888,1.520) | 0.274 |
| P for trend | 0.960(0.812,1.136) | 0.637 | 0.951(0.801,1.129) | 0.563 | 1.036(0.955,1.125) | 0.394 | 1.026(0.942,1.117) | 0.555 |
| **NO_2_** |  |  |  |  |  |  |  |  |
| 3 months before conception |  |  |  |  |  |  |  |  |
| Q 1 | Reference |  | Reference |  | Reference |  | Reference |  |
| Q 2 | 1.203(0.721,2.007) | 0.480 | 1.182(0.701,1.994) | 0.529 | 0.920(0.716,1.182) | 0.513 | 0.845(0.652,1.094) | 0.201 |
| Q 3 | 1.161(0.693,1.944) | 0.571 | 1.170(0.683,2.002) | 0.567 | 0.798(0.617,1.032) | 0.085 | 0.720(0.548,0.944) | 0.018* |
| Q 4 | 0.938(0.546,1.612) | 0.817 | 0.925(0.521,1.645) | 0.791 | 0.731(0.563,0.949) | 0.019* | 0.646(0.487,0.857) | 0.002* |
| P for trend | 0.979(0.832,1.153) | 0.802 | 0.975(0.819,1.162) | 0.781 | 0.898(0.827,0.975) | 0.010* | 0.863(0.788,0.944) | 0.001* |
| Trimester 1 |  |  |  |  |  |  |  |  |
| Q 1 | Reference |  | Reference |  | Reference |  | Reference |  |
| Q 2 | 1.249(0.745,2.094) | 0.400 | 1.240(0.737,2.084) | 0.417 | 1.188(0.921,1.533) | 0.184 | 1.184(0.914,1.533) | 0.201 |
| Q 3 | 1.006(0.585,1.728) | 0.984 | 1.015(0.588,1.751) | 0.958 | 1.028(0.793,1.334) | 0.833 | 1.030(0.790,1.343) | 0.825 |
| Q 4 | 1.203(0.715,2.024) | 0.487 | 1.234(0.708,2.149) | 0.458 | 0.887(0.679,1.159) | 0.380 | 0.913(0.685,1.216) | 0.533 |
| P for trend | 1.034(0.878,1.217) | 0.692 | 1.045(0.876,1.247) | 0.624 | 0.952(0.877,1.034) | 0.244 | 0.966(0.884,1.057) | 0.452 |
| Trimester 2 |  |  |  |  |  |  |  |  |
| Q 1 | Reference |  | Reference |  | Reference |  | Reference |  |
| Q 2 | 0.930(0.551,1.570) | 0.786 | 0.936(0.553,1.587) | 0.807 | 1.517(1.155,1.993) | 0.003* | 1.509(1.144,1.990) | 0.004* |
| Q 3 | 0.757(0.436,1.314) | 0.322 | 0.751(0.430,1.309) | 0.312 | 1.572(1.199,2.061) | 0.001* | 1.519(1.152,2.002) | 0.003* |
| Q 4 | 1.039(0.624,1.732) | 0.882 | 1.011(0.595,1.720) | 0.966 | 1.416(1.075,1.865) | 0.013* | 1.350(1.013,1.798) | 0.041* |
| P for trend | 0.994(0.840,1.176) | 0.941 | 0.982(0.824,1.171) | 0.840 | 1.104(1.016,1.199) | 0.019* | 1.092(1.000,1.192) | 0.049* |
| **CO** |  |  |  |  |  |  |  |  |
| 3 months before conception |  |  |  |  |  |  |  |  |
| Q 1 | Reference |  | Reference |  | Reference |  | Reference |  |
| Q 2 | 0.935(0.549,1.593) | 0.805 | 0.933(0.547,1.592) | 0.799 | 1.073(0.830,1.387) | 0.591 | 1.046(0.806,1.358) | 0.736 |
| Q 3 | 1.054(0.628,1.772) | 0.841 | 1.067(0.628,1.811) | 0.811 | 1.135(0.879,1.464) | 0.332 | 1.044(0.802,1.361) | 0.748 |
| Q 4 | 1.153(0.694,1.916) | 0.582 | 1.186(0.674,2.088) | 0.554 | 0.835(0.638,1.093) | 0.188 | 0.711(0.526,0.961) | 0.026* |
| P for trend | 1.057(0.898,1.245) | 0.503 | 1.065(0.887,1.278) | 0.501 | 0.957(0.881,1.038) | 0.290 | 0.913(0.832,1.002) | 0.055 |
| Trimester 1 |  |  |  |  |  |  |  |  |
| Q 1 | Reference |  | Reference |  | Reference |  | Reference |  |
| Q 2 | 1.197(0.717,1.998) | 0.491 | 1.199(0.716,2.006) | 0.490 | 1.380(1.067,1.785) | 0.014* | 1.416(1.091,1.839) | 0.009* |
| Q 3 | 1.004(0.590,1.710) | 0.987 | 1.009(0.588,1.729) | 0.975 | 1.155(0.886,1.504) | 0.286 | 1.142(0.871,1.497) | 0.337 |
| Q 4 | 1.080(0.640,1.822) | 0.773 | 1.091(0.623,1.910) | 0.760 | 0.999(0.762,1.311) | 0.996 | 1.047(0.781,1.402) | 0.759 |
| P for trend | 1.005(0.853,1.183) | 0.956 | 1.010(0.847,1.205) | 0.912 | 0.981(0.904,1.065) | 0.651 | 0.997(0.912,1.091) | 0.954 |
| Trimester 2 |  |  |  |  |  |  |  |  |
| Q 1 | Reference |  | Reference |  | Reference |  | Reference |  |
| Q 2 | 0.613(0.353,1.064) | 0.082 | 0.610(0.348,1.071) | 0.085 | 1.144(0.877,1.492) | 0.321 | 1.163(0.885,1.529) | 0.279 |
| Q 3 | 0.905(0.551,1.487) | 0.695 | 0.901(0.548,1.482) | 0.681 | 1.039(0.793,1.362) | 0.783 | 1.004(0.763,1.321) | 0.975 |
| Q 4 | 0.765(0.455,1.285) | 0.311 | 0.744(0.435,1.273) | 0.281 | 1.355(1.045,1.757) | 0.022* | 1.327(1.012,1.739) | 0.041* |
| P for trend | 0.951(0.804,1.126) | 0.561 | 0.937(0.787,1.115) | 0.462 | 1.087(1.001,1.181) | 0.048* | 1.074(0.984,1.171) | 0.109 |
| **O_3_** |  |  |  |  |  |  |  |  |
| 3 months before conception |  |  |  |  |  |  |  |  |
| Q 1 | Reference |  | Reference |  | Reference |  | Reference |  |
| Q 2 | 2.165(1.277,3.671) | 0.004* | 2.237(1.315,3.804) | 0.003* | 1.509(1.153,1.975) | 0.003* | 1.481(1.128,1.946) | 0.005* |
| Q 3 | 1.241(0.693,2.222) | 0.468 | 1.258(0.701,2.259) | 0.442 | 1.434(1.095,1.879) | 0.009* | 1.454(1.105,1.913) | 0.008 |
| Q 4 | 1.379(0.777,2.447) | 0.272 | 1.340(0.733,2.447) | 0.341 | 1.390(1.058,1.827) | 0.018* | 1.494(1.113,2.004) | 0.008* |
| P for trend | 1.017(0.864,1.198) | 0.836 | 1.023(0.859,1.218) | 0.800 | 1.090(1.004,1.184) | 0.040* | 1.127(1.029,1.234) | 0.010* |
| Trimester 1 |  |  |  |  |  |  |  |  |
| Q 1 | Reference |  | Reference |  | Reference |  | Reference |  |
| Q 2 | 0.719(0.409,1.266) | 0.253 | 0.717(0.405,1.270) | 0.254 | 1.590(1.207,2.095) | 0.001* | 1.596(1.205,2.113) | 0.001* |
| Q 3 | 1.503(0.936,2.413) | 0.091 | 1.513(0.931,2.461) | 0.095 | 1.753(1.337,2.298) | <0.001* | 1.747(1.323,2.307) | <0.001* |
| Q 4 | 0.817(0.474,1.409) | 0.468 | 0.814(0.462,1.432) | 0.475 | 1.503(1.138,1.984) | 0.004* | 1.514(1.132,2.024) | 0.005* |
| P for trend | 1.023(0.869,1.204) | 0.783 | 1.025(0.865,1.215) | 0.775 | 1.129(1.039,1.226) | 0.004* | 1.130(1.036,1.232) | 0.006* |
| Trimester 2 |  |  |  |  |  |  |  |  |
| Q 1 | Reference |  | Reference |  | Reference |  | Reference |  |
| Q 2 | 1.255(0.735,2.143) | 0.406 | 1.280(0.745,2.200) | 0.372 | 1.135(0.882,1.461) | 0.325 | 1.162(0.898,1.504) | 0.253 |
| Q 3 | 1.149(0.668,1.978) | 0.615 | 1.188(0.682,2.067) | 0.543 | 0.992(0.767,1.283) | 0.951 | 1.011(0.776,1.317) | 0.933 |
| Q 4 | 1.102(0.634,1.914 | 0.731 | 1.115(0.635,1.960) | 0.704 | 0.751(0.572,0.986) | 0.039* | 0.778(0.588,1.030) | 0.080 |
| P for trend | 1.018(0.860,1.206) | 0.832 | 1.022(0.860,1.214) | 0.803 | 0.911(0.838,0.989) | 0.027* | 0.919(0.844,1.001) | 0.053 |

Abbreviation: PM_2.5_: fine particulate matter; PM_10_: inhalable particulate matter; SO_2_: sulfur dioxide; NO_2_: nitrogen dioxide; CO: carbon monoxide; O_3_: ozone

The adjusted odds ratios (aOR) were calculated after controlling for maternal age, pre-pregnancy body mass index (BMI), primigravida and nulliparity status, use of assisted reproductive technology (ART), family history of hypertension, family history of hyperglycemia, presence of a scarred uterus and uterine fibroids, chorionicity, conception season, and mean temperature (Tmean).

**Table S6.** Ambient air pollution exposure and risk of twin pregnancy complete placenta previa and marginal placenta previa by window of exposure

|  | **Complete placenta previa** | | | **Marginal placenta previa** | | |
| --- | --- | --- | --- | --- | --- | --- |
|  | **3 months preconception** | **Trimester 1** | **Trimester 2** | **3 months preconception** | **Trimester 1** | **Trimester 2** |
|  | **aOR （95% CI）** | **aOR （95% CI）** | **aOR （95% CI）** | **aOR （95% CI）** | **aOR （95% CI）** | **aOR （95% CI）** |
| **PM2.5** | 0.997(0.964,1.028) | 0.996(0.967,1.026) | 0.982(0.955,1.000) | 0.977(0.945,1.008) | 0.975(0.950,1.000) | 0.988(0.962,1.014) |
| **PM10** | 0.996(0.972,1.020) | 0.997(0.976,1.019) | 0.988(0.968,1.008) | 0.983(0.960,1.006) | 0.982(0.963,1.000) | 0.992(0.972,1.011) |
| **SO2** | 0.987(0.835,1.164) | 0.977(0.837,1.144) | 0.912(0.789,1.047) | 0.868(0.742,1.011) | 0.883(0.774,1.006) | 0.922(0.807,1.051) |
| **NO2** | 0.962(0.839,1.098) | 1.000(0.894,1.126) | 0.947(0.860,1.042) | 0.869(0.769,0.979) | 0.947(0.861,1.044) | 0.933(0.854,1.021) |
| **CO** | 0.371(0.001,103.803) | 1.625(0.013,238.679) | 0.296(0.005,15.702) | 0.176(0.001,27.460) | 0.168(0.003,11.686) | 0.151(0.003,8.540) |
| **O3** | 0.988(0.764,1.292) | 0.981(0.789,1.206) | 0.985(0.827,1.156) | 1.171(0.925,1.498) | 0.98(0.807,1.175) | 1.080(0.929,1.242) |

Abbreviation: PM_2.5_: fine particulate matter; PM_10_: inhalable particulate matter; SO_2_: sulfur dioxide; NO2: nitrogen dioxide; CO: carbon monoxide; O3: ozone

The adjusted odds ratio (aOR) was controlled for maternal age, pre-pregnancy body mass index (BMI), primigravida and nulliparity status, assisted reproductive technology (ART) use, family history of hypertension, family history of hyperglycemia, presence of scarred uterus and uterine fibroids, chorionicity, conception season, and mean temperature (Tmean).

**Table S7.** Ambient air pollution exposure and risk of placenta previa and placenta accreta without previa in twin pregnancies among non-advanced maternal age women, by window of exposure

|  | **Placenta previa** | | | **Placenta accreta without previa** | | |
| --- | --- | --- | --- | --- | --- | --- |
|  | **3 months preconception** | **Trimester 1** | **Trimester 2** | **3 months preconception** | **Trimester 1** | **Trimester 2** |
|  | **aOR （95% CI）** | **aOR （95% CI）** | **aOR （95% CI）** | **aOR （95% CI）** | **aOR （95% CI）** | **aOR （95% CI）** |
| **PM2.5** | 1.007(0.985,1.028) | 1.002(0.982,1.021) | 1.157(0.736,1.839) | 1.040(1.029,1.051) | 0.995(0.984,1.005) | 0.986(0.976,0.997) |
| **PM10** | 1.004(0.988,1.020) | 1.002(0.987,1.016) | 0.998(0.983,1.013) | 1.030(1.022,1.038) | 0.996(0.988,1.003) | 0.989(0.981,0.996) |
| **SO2** | 1.021(0.911,1.143) | 1.020(0.919,1.132) | 0.993(0.893,1.103) | 1.273(1.201,1.349) | 0.996(0.944,1.051) | 0.916(0.868,0.966) |
| **NO2** | 1.029(0.944,1.121) | 1.003(0.928,1.084) | 0.984(0.916,1.059) | 1.174(1.124,1.228) | 0.999(0.956,1.044) | 0.954(0.920,0.990) |
| **CO** | 4.863(0.16,138.009) | 2.559(0.12,53.481) | 0.263(0.011,6.422) | 50.702(9.033,283.719) | 0.253(0.040,1.635) | 0.200(0.043,0.920) |
| **O3** | 0.981(0.823,1.173) | 1.006(0.867,1.163) | 1.022(0.901,1.150) | 0.840(0.768,0.919) | 1.043(0.963,1.128) | 1.036(0.974,1.101) |

Abbreviation: PM_2.5_: fine particulate matter; PM_10_: inhalable particulate matter; SO_2_: sulfur dioxide; NO_2_: nitrogen dioxide; CO: carbon monoxide; O_3_: ozone

The adjusted odds ratio (aOR) was adjusted for pre-pregnancy body mass index (BMI), primigravida and nulliparity status, assisted reproductive technology (ART) use, family history of hypertension, family history of hyperglycemia, presence of scarred uterus and uterine fibroids, chorionicity, conception season, and mean temperature (Tmean).

**Table S8.** Ambient air pollution exposure and the risk of placenta previa and placenta accreta without previa in nulliparity women with twin pregnancies, by exposure window.

|  | **Placenta previa** | | | **Placenta accreta without previa** | | |
| --- | --- | --- | --- | --- | --- | --- |
|  | **3 months preconception** | **Trimester 1** | **Trimester 2** | **3 months preconception** | **Trimester 1** | **Trimester 2** |
|  | **aOR （95% CI）** | **aOR （95% CI）** | **aOR （95% CI）** | **aOR （95% CI）** | **aOR （95% CI）** | **aOR （95% CI）** |
| **PM2.5** | 0.984(0.959,1.007) | 1.012(0.991,1.033) | 1.022(1.001,1.043) | 0.990(0.977,1.002) | 1.003(0.993,1.014) | 0.996(0.985,1.006) |
| **PM10** | 0.985(0.967,1.003) | 1.009(0.994,1.025) | 1.017(1.002,1.033) | 0.994(0.985,1.003) | 1.002(0.994,1.010) | 0.997(0.989,1.004) |
| **SO2** | 0.880(0.776,0.996) | 1.045(0.933,1.174) | 1.123(1.008,1.252) | 0.975(0.917,1.037) | 1.034(0.976,1.096) | 0.999(0.947,1.053) |
| **NO2** | 0.914(0.828,1.007) | 1.054(0.966,1.153) | 1.064(0.986,1.151) | 0.998(0.951,1.047) | 1.019(0.976,1.065) | 1.004(0.968,1.042) |
| **CO** | 0.435(0.006,26.467) | 3.835(0.102,157.418) | 32.352(1.152,1002.749) | 2.204(0.273,17.540) | 2.276(0.356,14.799) | 0.857(0.172,4.291) |
| **O3** | 1.010(0.828,1.237) | 0.989(0.841,1.155) | 0.924(0.805,1.049) | 0.992(0.900,1.095) | 1.024(0.944,1.109) | 0.999(0.938,1.061) |

Abbreviation: PM_2.5_: fine particulate matter; PM_10_: inhalable particulate matter; SO_2_: sulfur dioxide; NO_2_: nitrogen dioxide; CO: carbon monoxide; O_3_: ozone

The adjusted odds ratios (aOR) were controlled for maternal age, pre-pregnancy body mass index (BMI), assisted reproductive technology (ART) use, family history of hypertension, family history of hyperglycemia, presence of a scarred uterus and uterine fibroids, chorionicity, conception season, and mean temperature (Tmean).

**Table S9.** Ambient air pollution exposure and risk of placenta previa and placenta accreta without previa in twin pregnancies without assisted reproductive technology (ART), by exposure window.

|  | **Placenta previa** | | | **Placenta accreta without previa** | | |
| --- | --- | --- | --- | --- | --- | --- |
|  | **3 months preconception** | **Trimester 1** | **Trimester 2** | **3 months preconception** | **Trimester 1** | **Trimester 2** |
|  | **aOR （95% CI）** | **aOR （95% CI）** | **aOR （95% CI）** | **aOR （95% CI）** | **aOR （95% CI）** | **aOR （95% CI）** |
| **PM2.5** | 1.001(0.952,1.050) | 1.008(0.969,1.049) | 1.002(0.960,1.044) | 1.011(0.987,1.036) | 1.023(1.002,1.045) | 1.010(0.989,1.030) |
| **PM10** | 0.997(0.960,1.033) | 1.006(0.977,1.037) | 1.004(0.973,1.036) | 1.009(0.991,1.027) | 1.018(1.002,1.034) | 1.007(0.992,1.023) |
| **SO2** | 0.933(0.720,1.199) | 1.038(0.842,1.290) | 1.065(0.856,1.330) | 1.023(0.902,1.161) | 1.112(0.996,1.245) | 1.069(0.962,1.190) |
| **NO2** | 0.989(0.815,1.196) | 1.024(0.885,1.188) | 1.020(0.878,1.196) | 1.053(0.956,1.160) | 1.047(0.965,1.139) | 1.044(0.968,1.128) |
| **CO** | 1.949(0.001,5855.642) | 5.56(0.014,2082.075) | 0.204(0.00,164.403) | 3.983(0.064,239.515) | 6.27(0.182,232.989) | 5.268(0.195,151.962) |
| **O3** | 0.905(0.617,1.338) | 1.025(0.765,1.348) | 1.053(0.815,1.325) | 0.974(0.811,1.174) | 0.984(0.843,1.142) | 0.924(0.807,1.049) |

Abbreviation: PM_2.5_: fine particulate matter; PM_10_: inhalable particulate matter; SO_2_: sulfur dioxide; NO_2_: nitrogen dioxide; CO: carbon monoxide; O_3_: ozone

The adjusted odds ratios (aOR) were controlled for maternal age, pre-pregnancy body mass index (BMI), primigravida and nulliparity status, family history of hypertension, family history of hyperglycemia, presence of a scarred uterus and uterine fibroids, chorionicity, conception season, and mean temperature (Tmean).

**Table S10.** Ambient air pollution exposure and risk of placenta previa and placenta accreta without previa in twin pregnancies without scarred uterus and uterine fibroids, by exposure window.

|  | **Placenta previa** | | | **Placenta accreta without previa** | | |
| --- | --- | --- | --- | --- | --- | --- |
|  | **3 months preconception** | **Trimester 1** | **Trimester 2** | **3 months preconception** | **Trimester 1** | **Trimester 2** |
|  | **aOR （95% CI）** | **aOR （95% CI）** | **aOR （95% CI）** | **aOR （95% CI）** | **aOR （95% CI）** | **aOR （95% CI）** |
| **PM2.5** | 1.008(0.985,1.031) | 0.997(0.977,1.018) | 0.997(0.976,1.018) | 0.983(0.971,0.994) | 0.979(0.969,0.990) | 0.988(0.978,0.998) |
| **PM10** | 1.006(0.989,1.024) | 0.998(0.983,1.013) | 0.998(0.982,1.013) | 0.986(0.977,0.995) | 0.985(0.977,0.993) | 0.992(0.985,0.999) |
| **SO2** | 1.027(0.911,1.158) | 0.980(0.879,1.093) | 0.995(0.894,1.107) | 0.880(0.828,0.935) | 0.887(0.838,0.938) | 0.943(0.895,0.993) |
| **NO2** | 1.009(0.919,1.107) | 0.973(0.899,1.056) | 0.985(0.916,1.062) | 0.864(0.822,0.907) | 0.927(0.889,0.966) | 0.974(0.94,1.009) |
| **CO** | 2.025(0.035,109.437) | 0.135(0.004,4.376) | 0.630(0.024,17.039) | 0.008(0.001,0.064) | 0.146(0.024,0.884) | 0.997(0.205,4.873) |
| **O3** | 1.047(0.868,1.271) | 1.092(0.941,1.259) | 1.066(0.943,1.197) | 1.295(1.172,1.434) | 1.059(0.981,1.142) | 0.987(0.927,1.049) |

Abbreviation: PM_2.5_: fine particulate matter; PM_10_: inhalable particulate matter; SO_2_: sulfur dioxide; NO_2_: nitrogen dioxide; CO: carbon monoxide; O_3_: ozone

The adjusted odds ratios (aOR) were controlled for maternal age, pre-pregnancy body mass index (BMI), primigravida and nulliparity status, use of assisted reproductive technology (ART), family history of hypertension, family history of hyperglycemia, chorionicity, conception season, and mean temperature (Tmean).

**Table S11.** The risk factor analysis of complete placenta previa and marginal placenta previa in twin pregnancies during three exposure windows: 3 months before conception, Trimester 1, and Trimester 2.

| **EC** | **Complete placenta previa** | | | | **Marginal placenta previa** | | | |
| --- | --- | --- | --- | --- | --- | --- | --- | --- |
|  | **Unadjusted Model** | | **Adjusted Model** | | **Unadjusted Model** | | **Adjusted Model** | |
|  | **OR (95% CI)** | ***p*-value** | **aOR (95% CI)** | ***p*-value** | **OR (95% CI)** | ***p*-value** | **aOR (95% CI)** | ***p*-value** |
| **PM2.5** |  |  |  |  |  |  |  |  |
| 3 months before conception |  |  |  |  |  |  |  |  |
| Q 1 | Reference |  | Reference |  | Reference |  | Reference |  |
| Q 2 | 1.984(0.887,4.441) | 0.095 | 1.900(0.835,4.322) | 0.126 | 1.968(1.025,3.779) | 0.042* | 2.110(1.084,4.105) | 0.028* |
| Q 3 | 1.135(0.459,2.807) | 0.784 | 1.107(0.446,2.749) | 0.827 | 0.857(0.394,1.864) | 0.697 | 0.922(0.422,2.015) | 0.839 |
| Q 4 | 1.564(0.674,3.633) | 0.298 | 1.551(0.631,3.810) | 0.339 | 0.785(0.354,1.738) | 0.550 | 0.945(0.406,2.199) | 0.896 |
| P for trend | 1.061(0.829,1.359) | 0.637 | 1.048(0.806,1.362) | 0.728 | 0.858(0.686,1.072) | 0.178 | 0.896(0.707,1.135) | 0.361 |
| Trimester 1 |  |  |  |  |  |  |  |  |
| Q 1 | Reference |  | Reference |  | Reference |  | Reference |  |
| Q 2 | 1.074(0.515,2.238) | 0.849 | 1.067(0.510,2.230) | 0.864 | 1.297(0.684,2.459) | 0.426 | 1.334(0.700,2.542) | 0.380 |
| Q 3 | 0.564(0.236,1.352) | 0.199 | 0.575(0.238,1.386) | 0.217 | 0.704(0.334,1.483) | 0.356 | 0.682(0.322,1.446) | 0.318 |
| Q 4 | 1.007(0.477,2.124) | 0.986 | 1.072(0.483,2.378) | 0.864 | 0.762(0.368,1.578) | 0.465 | 0.675(0.319,1.428) | 0.304 |
| P for trend | 0.947(0.739,1.213) | 0.666 | 0.961(0.739,1.250) | 0.765 | 0.870(0.696,1.087) | 0.219 | 0.839(0.668,1.055) | 0.134 |
| Trimester 2 |  |  |  |  |  |  |  |  |
| Q 1 | Reference |  | Reference |  | Reference |  | Reference |  |
| Q 2 | 0.874(0.424,1.802) | 0.715 | 0.837(0.399,1.754) | 0.637 | 0.902(0.481,1.689) | 0.747 | 0.981(0.516,1.865) | 0.952 |
| Q 3 | 0.619(0.279,1.371) | 0.237 | 0.611(0.274,1.360) | 0.227 | 0.327(0.138,0.772) | 0.011* | 0.330(0.139,0.782) | 0.012* |
| Q 4 | 0.691(0.319,1.497) | 0.348 | 0.628(0.278,1.418) | 0.263 | 0.808(0.423,1.541) | 0.517 | 0.940(0.478,1.847) | 0.857 |
| P for trend | 0.861(0.670,1.105) | 0.240 | 0.838(0.648,1.084) | 0.178 | 0.858(0.686,1.072) | 0.178 | 0.879(0.699,1.106) | 0.272 |
| **PM10** |  |  |  |  |  |  |  |  |
| 3 months before conception |  |  |  |  |  |  |  |  |
| Q 1 | Reference |  | Reference |  | Reference |  | Reference |  |
| Q 2 | 2.027(0.906,4.537) | 0.086 | 1.887(0.829,4.298) | 0.130 | 1.150(0.618,2.140) | 0.659 | 1.264(0.668,2.390) | 0.471 |
| Q 3 | 1.120(0.453,2.769) | 0.806 | 1.083(0.436,2.692) | 0.864 | 0.635(0.306,1.316) | 0.222 | 0.692(0.332,1.442) | 0.325 |
| Q 4 | 1.573(0.677,3.653) | 0.292 | 1.522(0.622,3.725) | 0.358 | 0.575(0.272,1.215) | 0.147 | 0.679(0.306,1.506) | 0.341 |
| P for trend | 1.059(0.827,1.357) | 0.648 | 1.041(0.801,1.352) | 0.765 | 0.806(0.643,1.009) | 0.060 | 0.840(0.663,1.065) | 0.150 |
| Trimester 1 |  |  |  |  |  |  |  |  |
| Q 1 | Reference |  | Reference |  | Reference |  | Reference |  |
| Q 2 | 1.438(0.683,3.028) | 0.340 | 1.403(0.664,2.963) | 0.375 | 1.319(0.696,2.501) | 0.396 | 1.420(0.744,2.709) | 0.288 |
| Q 3 | 0.666(0.271,1.637) | 0.376 | 0.670(0.271,1.654) | 0.385 | 0.643(0.300,1.381) | 0.258 | 0.648(0.300,1.397) | 0.268 |
| Q 4 | 1.179(0.542,2.562) | 0.678 | 1.242(0.549,2.811) | 0.603 | 0.832(0.408,1.698) | 0.613 | 0.758(0.365,1.574) | 0.457 |
| P for trend | 0.978(0.764,1.252) | 0.861 | 0.994(0.766,1.291) | 0.966 | 0.883(0.706,1.102) | 0.271 | 0.861(0.687,1.079) | 0.195 |
| Trimester 2 |  |  |  |  |  |  |  |  |
| Q 1 | Reference |  | Reference |  | Reference |  | Reference |  |
| Q 2 | 0.936(0.460,1.906) | 0.856 | 0.923(0.449,1.897) | 0.828 | 0.998(0.533,1.867) | 0.994 | 1.035(0.549,1.952) | 0.916 |
| Q 3 | 0.624(0.281,1.382) | 0.245 | 0.605(0.271,1.347) | 0.219 | 0.394(0.173,0.900) | 0.027* | 0.399(0.174,0.914) | 0.030* |
| Q 4 | 0.626(0.282,1.386) | 0.248 | 0.567(0.248,1.297) | 0.179 | 0.796(0.410,1.547) | 0.502 | 0.899(0.452,1.788) | 0.761 |
| P for trend | 0.834(0.649,1.071) | 0.155 | 0.808(0.625,1.045) | 0.104 | 0.858(0.686,1.072) | 0.177 | 0.879(0.698,1.106) | 0.270 |
| **SO2** |  |  |  |  |  |  |  |  |
| 3 months before conception |  |  |  |  |  |  |  |  |
| Q 1 | Reference |  | Reference |  | Reference |  | Reference |  |
| Q 2 | 1.710(0.803,3.640) | 0.164 | 1.640(0.743,3.621) | 0.221 | 0.941(0.506,1.748) | 0.847 | 1.030(0.542,1.956) | 0.928 |
| Q 3 | 0.925(0.391,2.190) | 0.860 | 0.912(0.382,2.173) | 0.835 | 0.566(0.277,1.157) | 0.119 | 0.614(0.299,1.262) | 0.185 |
| Q 4 | 1.133(0.497,2.582) | 0.766 | 1.102(0.456,2.660) | 0.830 | 0.529(0.254,1.104) | 0.090 | 0.616(0.282,1.346) | 0.225 |
| P for trend | 0.970(0.758,1.242) | 0.810 | 0.948(0.731,1.230) | 0.687 | 0.787(0.627,0.988) | 0.039* | 0.818(0.646,1.037) | 0.096 |
| Trimester 1 |  |  |  |  |  |  |  |  |
| Q 1 | Reference |  | Reference |  | Reference |  | Reference |  |
| Q 2 | 0.815(0.379,1.750) | 0.599 | 0.824(0.380,1.784) | 0.623 | 1.018(0.535,1.936) | 0.956 | 1.001(0.525,1.911) | 0.996 |
| Q 3 | 0.802(0.373,1.723) | 0.571 | 0.823(0.381,1.776) | 0.619 | 0.686(0.337,1.397) | 0.299 | 0.681(0.333,1.394) | 0.293 |
| Q 4 | 0.810(0.377,1.740) | 0.589 | 0.845(0.372,1.922) | 0.688 | 0.693(0.340,1.411) | 0.312 | 0.608(0.292,1.270) | 0.186 |
| P for trend | 0.934(0.729,1.196) | 0.588 | 0.946(0.729,1.228) | 0.677 | 0.862(0.690,1.077) | 0.192 | 0.833(0.663,1.046) | 0.115 |
| Trimester 2 |  |  |  |  |  |  |  |  |
| Q 1 | Reference |  | Reference |  | Reference |  | Reference |  |
| Q 2 | 0.766(0.370,1.586) | 0.472 | 0.760(0.365,1.582) | 0.463 | 1.015(0.542,1.899) | 0.963 | 1.043(0.555,1.959) | 0.897 |
| Q 3 | 0.594(0.270,1.304) | 0.194 | 0.585(0.265,1.292) | 0.185 | 0.298(0.119,0.746) | 0.010* | 0.303(0.121,0.759) | 0.011 |
| Q 4 | 0.648(0.302,1.391) | 0.265 | 0.600(0.273,1.317) | 0.203 | 0.906(0.476,1.724) | 0.764 | 1.010(0.523,1.9500 | 0.977 |
| P for trend | 0.848(0.661,1.089) | 0.197 | 0.829(0.643,1.069) | 0.147 | 0.882(0.706,1.102) | 0.269 | 0.904(0.721,1.134) | 0.383 |
| **NO2** |  |  |  |  |  |  |  |  |
| 3 months before conception |  |  |  |  |  |  |  |  |
| Q 1 | Reference |  | Reference |  | Reference |  | Reference |  |
| Q 2 | 1.466(0.677,3.177) | 0.332 | 1.392(0.632,3.068) | 0.412 | 0.823(0.445,1.522) | 0.535 | 0.869(0.465,1.624) | 0.660 |
| Q 3 | 1.164(0.519,2.611) | 0.713 | 1.062(0.457,2.470) | 0.889 | 0.473(0.229,0.976) | 0.043* | 0.510(0.241,1.080) | 0.079 |
| Q 4 | 1.023(0.441,2.372) | 0.958 | 0.947(0.388,2.315) | 0.905 | 0.473(0.229,0.976) | 0.043* | 0.523(0.242,1.131) | 0.100 |
| P for trend | 0.981(0.765,1.257) | 0.879 | 0.951(0.726,1.246) | 0.715 | 0.752(0.598,0.945) | 0.014* | 0.778(0.609,0.994) | 0.044* |
| Trimester 1 |  |  |  |  |  |  |  |  |
| Q 1 | Reference |  | Reference |  | Reference |  | Reference |  |
| Q 2 | 1.244(0.579,2.674) | 0.575 | 1.226(0.568,2.647) | 0.603 | 1.351(0.673,2.711) | 0.397 | 1.456(0.719,2.947) | 0.297 |
| Q 3 | 1.008(0.450,2.256) | 0.985 | 1.027(0.456,2.316) | 0.948 | 1.374(0.685,2.758) | 0.371 | 1.353(1.353) | 0.398 |
| Q 4 | 1.002(0.448,2.243) | 0.996 | 1.036(0.437,2.459) | 0.936 | 0.855(0.393,1.859) | 0.693 | 0.734(0.329,1.638) | 0.451 |
| P for trend | 0.979(0.764,1.253) | 0.864 | 0.996(0.761,1.303) | 0.976 | 0.964(0.773,1.203) | 0.746 | 0.927(0.737,1.166) | 0.518 |
| Trimester 2 |  |  |  |  |  |  |  |  |
| Q 1 | Reference |  | Reference |  | Reference |  | Reference |  |
| Q 2 | 0.796(0.371,1.711) | 0.560 | 0.814(0.376,1.762) | 0.601 | 0.906(0.484,1.6970 | 0.757 | 0.906(0.481,1.7080 | 0.761 |
| Q 3 | 0.861(0.407,1.820) | 0.695 | 0.814(0.381,1.741) | 0.596 | 0.422(0.192,0.926) | 0.031* | 0.434(0.197,0.958) | 0.039* |
| Q 4 | 0.733(0.335,1.606)0.438 | 0.438 | 0.685(0.304,1.544) | 0.361 | 0.713(0.365,1.391) | 0.321 | 0.792(0.395,1.5860 | 0.510 |
| P for trend | 0.917(0.715,1.175) | 0.493 | 0.890(0.687,1.154) | 0.380 | 0.836(0.668,1.046) | 0.117 | 0.861(0.682,1.087) | 0.209 |
| **CO** |  |  |  |  |  |  |  |  |
| 3 months before conception |  |  |  |  |  |  |  |  |
| Q 1 | Reference |  | Reference |  | Reference |  | Reference |  |
| Q 2 | 1.178(0.525,2.643) | 0.691 | 1.239(0.550,2.791) | 0.605 | 0.544(0.267,1.105) | 0.092 | 0.329(0.275,1.142) | 0.111 |
| Q 3 | 1.008(0.435,2.337) | 0.985 | 1.037(0.439,2.450) | 0.934 | 0.690(0.356,1.340) | 0.273 | 0.758(0.387,1.485) | 0.420 |
| Q 4 | 1.463(0.675,3.170) | 0.335 | 1.464(0.617,3.473) | 0.387 | 0.683(0.352,1.324) | 0.259 | 0.859(0.409,1.805) | 0.688 |
| P for trend | 1.111(0.867,1.424) | 0.407 | 1.104(0.836,1.459) | 0.486 | 0.895(0.717,1.118) | 0.329 | 0.948(0.742,1.212) | 0.672 |
| Trimester 1 |  |  |  |  |  |  |  |  |
| Q 1 | Reference |  | Reference |  | Reference |  | Reference |  |
| Q 2 | 1.171(0.538,2.545) | 0.691 | 1.179(0.540,2.575) | 0.680 | 1.473(0.723,3.001) | 0.286 | 1.528(0.746,3.129) | 0.247 |
| Q 3 | 0.915(0.401,2.084) | 0.832 | 0.929(0.402,2.148） | 0.863 | 1.154(0.546,2.439) | 0.708 | 1.113(0.523,2.368) | 0.780 |
| Q 4 | 1.172(0.539,2.548) | 0.689 | 1.261(0.540,2.948) | 0.592 | 1.317(0.636,2.727) | 0.459 | 1.160(0.544,2.474) | 0.700 |
| P for trend | 1.025(0.800,1.312) | 0.847 | 1.046(0.797,1.374) | 0.744 | 1.053(0.844,1.314) | 0.650 | 1.012(0.803,1.276) | 0.916 |
| Trimester 2 |  |  |  |  |  |  |  |  |
| Q 1 | Reference |  | Reference |  | Reference |  | Reference |  |
| Q 2 | 1.349(0.634,2.868) | 0.437 | 1.394(0.643,3.018) | 0.400 | 0.805(0.422,1.536) | 0.511 | 0.760(0.392,1.472) | 0.416 |
| Q 3 | 0.913(0.401,2.079) | 0.827 | 0.902(0.395,2.063) | 0.807 | 0.661(0.334,1.309) | 0.235 | 0.641(0.323,1.275) | 0.205 |
| Q 4 | 1.009(0.451,2.259) | 0.982 | 0.933(0.405,2.145) | 0.869 | 0.567(0.277,1.159) | 0.120 | 0.623(0.299,1.301) | 0.208 |
| P for trend | 0.963(0.752,1.233) | 0.763 | 0.946(0.732,1.222) | 0.669 | 0.825(0.659,1.033) | 0.094 | 0.843(0.668,1.063) | 0.149 |
| **O3** |  |  |  |  |  |  |  |  |
| 3 months before conception |  |  |  |  |  |  |  |  |
| Q 1 | Reference |  | Reference |  | Reference |  | Reference |  |
| Q 2 | 1.149(0.558,2.369) | 0.706 | 1.132(0.546,2.350) | 0.739 | 0.817(0.391,1.709) | 0.591 | 0.821(0.391,1.722) | 0.601 |
| Q 3 | 0.423(0.162,1.105) | 0.079 | 0.443(0.168,1.165) | 0.099 | 0.740(0.348,1.573) | 0.434 | 0.718(0.336,1.532) | 0.391 |
| Q 4 | 1.111(0.533,2.316) | 0.778 | 1.169(0.526,2.598) | 0.702 | 1.491(0.782,2.841) | 0.225 | 1.336(0.670,2.666) | 0.411 |
| P for trend | 0.955(0.745,1.224) | 0.714 | 0.966(0.740,1.262) | 0.800 | 1.145(0.916,1.431) | 0.235 | 1.086(0.858,1.375) | 0.494 |
| Trimester 1 |  |  |  |  |  |  |  |  |
| Q 1 | Reference |  | Reference |  | Reference |  | Reference |  |
| Q 2 | 0.767(0.334,1.757) | 0.530 | 0.734(0.316,1.702) | 0.471 | 1.095(0.561,2.139) | 0.790 | 1.132(0.575,2.232) | 0.719 |
| Q 3 | 1.048(0.483,2.273) | 0.906 | 0.965(0.437,2.130) | 0.929 | 1.128(0.582,2.184) | 0.722 | 1.233(0.628,2.423) | 0.543 |
| Q 4 | 1.179(0.558,2.491) | 0.667 | 1.143(0.520,2.513) | 0.739 | 0.601(0.274,1.319) | 0.204 | 0.660(0.294,1.479) | 0.313 |
| P for trend | 1.085(0.848,1.389) | 0.517 | 1.074(0.827,1.394) | 0.592 | 0.886(0.709,1.106) | 0.285 | 0.916(0.728,1.152) | 0.452 |
| Trimester 2 |  |  |  |  |  |  |  |  |
| Q 1 | Reference |  | Reference |  | Reference |  | Reference |  |
| Q 2 | 1.097(0.482,2.500) | 0.825 | 1.148(0.499,2.637) | 0.746 | 1.629(0.765,3.469) | 0.206 | 1.627(0.758,3.493) | 0.212 |
| Q 3 | 1.535(0.715,3.296) | 0.272 | 1.593(0.731,3.472) | 0.241 | 1.459(0.674,3.162) | 0.338 | 1.416(0.646,3.102) | 0.385 |
| Q 4 | 1.017(0.439,2.359) | 0.968 | 1.080(0.458,2.545) | 0.861 | 1.763(0.834,3.726) | 0.138 | 1.673(0.781,3.583) | 0.185 |
| P for trend | 1.044(0.815,1.338) | 0.732 | 1.061(0.823,1.367) | 0.649 | 1.157(0.925,1.447) | 0.201 | 1.134(0.903,1.423) | 0.278 |

*p < 0.05

Abbreviation: PM_2.5_: fine particulate matter; PM_10_: inhalable particulate matter; SO_2_: sulfur dioxide; NO_2_: nitrogen dioxide; CO: carbon monoxide; O_3_: ozone

The adjusted odds ratios (aOR) were controlled for maternal age, pre-pregnancy body mass index (BMI), primigravida and nulliparity status, assisted reproductive technology (ART) use, family history of hypertension, family history of hyperglycemia, presence of a scarred uterus and uterine fibroids, chorionicity, conception season, and mean temperature (Tmean).

**Table S12**. In the dual-pollutant model, the relationship between exposure to ambient air pollution for 3 months preconception and placenta previa as well as placenta accreta without previa in twin pregnancies.

|  | **Placenta previa** | **Placenta accreta without previa** |
| --- | --- | --- |
| **PM2.5 adjusted for NO2** | 0.977(0.819,1.171) | 0.993(0.971,1.015) |
| **PM2.5 adjusted for CO** | 0.991(0.961,1.022) | 0.977(0.962,0.993) |
| **PM2.5 adjusted for O3** | 0.998(0.975,1.021) | 0.982(0.970,0.994) |
| **PM10 adjusted for NO2** | 1.006(0.973,1.040) | 1.001(0.984,1.019) |
| **PM10 adjusted for CO** | 0.995(0.974,1.018) | 0.986(0.975,0.998) |
| **PM10 adjusted for O3** | 1.000(0.983,1.017) | 0.988(0.980,0.997) |
| **SO2 adjusted for CO** | 0.970(0.836,1.127) | 0.885(0.819,0.956) |
| **SO2 adjusted for O3** | 0.997(0.885,1.121) | 0.907(0.853,0.964) |
| **NO2 adjusted for PM2.5** | 0.977(0.819,1.171) | 0.936(0.856,1.023) |
| **NO2 adjusted for PM10** | 0.954(0.795,1.149) | 0.907(0.827,0.995) |
| **NO2 adjusted for CO** | 0.937(0.808,1.087) | 0.876(0.811,0.946) |
| **NO2 adjusted for O3** | 0.989(0.886,1.102) | 0.921(0.871,0.974) |
| **CO adjusted for PM2.5** | 4.239(0.019,977.116) | 1.509(0.097,23.509) |
| **CO adjusted for PM10** | 2.816(0.014,606.535) | 0.898(0.059,13.708) |
| **CO adjusted for SO2** | 2.603(0.017,452.195) | 1.456(0.105,20.717) |
| **CO adjusted for NO2** | 12.647(0.022,8152.726) | 9.024(0.331,252.605) |
| **CO adjusted for O3** | 3.531(0.027,440.85) | 0.323(0.026,3.973) |
| **O3 adjusted for PM2.5** | 1.029(0.854,1.244) | 1.080(1.000,1.192) |
| **O3 adjusted for PM10** | 1.034(0.859,1.249) | 1.088(1.004,1.200) |
| **O3 adjusted for SO2** | 1.033(0.858,1.249) | 1.074(0.974,1.186) |
| **O3 adjusted for NO2** | 1.022(0.823,1.272) | 1.029(0.918,1.155) |
| **O3 adjusted for CO** | 1.076(0.858,1.359) | 1.101(1.008,1.243) |

Abbreviation: PM_2.5_: fine particulate matter; PM_10_: inhalable particulate matter; SO_2_: sulfur dioxide; NO_2_: nitrogen dioxide; CO: carbon monoxide; O_3_: ozone

The adjusted odds ratio (aOR) was adjusted not only for the corresponding other pollutants but also for maternal age, pre-pregnancy body mass index (BMI), primigravida and nulliparity status, assisted reproductive technology (ART) use, family history of hypertension, family history of hyperglycemia, presence of a scarred uterus and uterine fibroids, chorionicity, conception season, and mean temperature (Tmean).

**Table S13**. In the dual-pollutant model, the relationship between exposure to ambient air pollution for trimester1 and placenta previa as well as placenta accreta without previa in twin pregnancies.

|  | **Placenta previa** | **Placenta accreta without previa** |
| --- | --- | --- |
| **PM2.5 adjusted for NO2** | 1.005(0.966,1.046) | 0.993(0.971,1.016) |
| **PM2.5 adjusted for CO** | 1.013(0.981,1.048) | 0.977(0.962,0.993) |
| **PM2.5 adjusted for O3** | 1.005(0.983,1.028) | 0.982(0.970,0.994) |
| **PM10 adjusted for CO** | 1.009(0.985,1.035) | 0.986(0.975,0.998) |
| **PM10 adjusted for O3** | 1.004(0.987,1.020) | 0.988(0.980,0.997) |
| **SO2 adjusted for CO** | 1.073(0.905,1.274) | 0.886(0.82,0.958) |
| **SO2 adjusted for O3** | 1.031(0.912,1.165) | 0.907(0.853,0.964) |
| **NO2 adjusted for PM2.5** | 1.008(0.860,1.189) | 0.934(0.855,1.022) |
| **NO2 adjusted for CO** | 1.067(0.912,1.247) | 0.877(0.812,0.947) |
| **NO2 adjusted for O3** | 1.019(0.907,1.142) | 0.921(0.870,0.974) |
| **CO adjusted for PM2.5** | 0.282(0.001,78.837) | 1.381(0.089,21.609) |
| **CO adjusted for PM10** | 0.308(0.001,89.472) | 0.821(0.054,12.583) |
| **CO adjusted for SO2** | 0.304(0.001,70.67) | 1.328(0.095,18.966) |
| **CO adjusted for NO2** | 0.168(0.0,131.423) | 8.216(0.3,230.747) |
| **CO adjusted for O3** | 0.670(0.006,69.898) | 0.306(0.024,3.766) |
| **O3 adjusted for PM2.5** | 0.973(0.822,1.148) | 1.082(1.007,1.194) |
| **O3 adjusted for PM10** | 0.971(0.821,1.146) | 1.090(1.018,1.203) |
| **O3 adjusted for SO2** | 0.974(0.822,1.153) | 1.077(0.976,1.189) |
| **O3 adjusted for NO2** | 0.977(0.790,1.207) | 1.032(0.920,1.157) |
| **O3 adjusted for CO** | 0.941(0.770,1.152) | 1.101(1.011,1.244) |

Abbreviation: PM_2.5_: fine particulate matter; PM_10_: inhalable particulate matter; SO_2_: sulfur dioxide; NO_2_: nitrogen dioxide; CO: carbon monoxide; O_3_: ozone

The adjusted odds ratio (aOR) was adjusted for the corresponding other pollutants, as well as for maternal age, pre-pregnancy body mass index (BMI), primigravida and nulliparity status, assisted reproductive technology (ART) use, family history of hypertension, family history of hyperglycemia, scarred uterus and uterine fibroids, chorionicity, conception season, and mean temperature (Tmean).

**Table S14**. In the dual-pollutant model, the relationship between exposure to ambient air pollution for trimester2 and placenta previa as well as placenta accreta without previa in twin pregnancies.

|  | **Placenta previa** | **Placenta accreta without previa** |
| --- | --- | --- |
| **PM2.5 adjusted for NO2** | 0.981(0.941,1.023) | 0.964(0.945,0.984) |
| **PM2.5 adjusted for CO** | 0.983(0.950,1.018) | 0.971(0.955,0.987) |
| **PM2.5 adjusted for O3** | 0.986(0.963,1.010) | 0.991(0.979,1.003) |
| **PM10 adjusted for NO2** | 0.981(0.952,1.012) | 0.976(0.961,0.991) |
| **PM10 adjusted for CO** | 0.984(0.960,1.010) | 0.980(0.968,0.993) |
| **PM10 adjusted for O3** | 0.989(0.971,1.006) | 0.994(0.986,1.003) |
| **SO2 adjusted for CO** | 0.904(0.756,1.081) | 0.952(0.871,1.042) |
| **SO2 adjusted for O3** | 0.924(0.814,1.047) | 1.002(0.942,1.067) |
| **NO2 adjusted for PM2.5** | 1.037(0.895,1.204) | 1.149(1.066,1.239) |
| **NO2 adjusted for PM10** | 1.057(0.912,1.227) | 1.137(1.055,1.226) |
| **NO2 adjusted for O3** | 0.948(0.840,1.067) | 1.017(0.958,1.078) |
| **CO adjusted for PM2.5** | 3.788(0.016,817.862) | 162.706(11.041,2391.686) |
| **CO adjusted for PM10** | 6.332(0.028,1322.796) | 117.653(7.86,1758.888) |
| **CO adjusted for SO2** | 5.372(0.022,1332.481) | 11.234(0.712,179.434) |
| **CO adjusted for O3** | 0.175(0.001,24.004) | 4.134(0.375,45.423) |
| **O3 adjusted for PM2.5** | 0.966(0.840,1.109) | 0.937(0.871,1.007) |
| **O3 adjusted for PM10** | 0.962(0.838,1.102) | 0.943(0.878,1.013) |
| **O3 adjusted for SO2** | 0.958(0.829,1.104) | 0.972(0.902,1.047) |
| **O3 adjusted for NO2** | 0.944(0.776,1.147) | 0.993(0.898,1.098) |
| **O3 adjusted for CO** | 0.964(0.802,1.159) | 1.014(0.923,1.114) |

Abbreviation: PM_2.5_: fine particulate matter; PM_10_: inhalable particulate matter; SO_2_: sulfur dioxide; NO_2_: nitrogen dioxide; CO: carbon monoxide; O_3_: ozone

The adjusted odds ratio (aOR) was adjusted for the corresponding other pollutants, as well as for maternal age, pre-pregnancy body mass index (BMI), primigravida and nulliparity status, assisted reproductive technology (ART) use, family history of hypertension, family history of hyperglycemia, scarred uterus and uterine fibroids, chorionicity, conception season, and mean temperature (Tmean).

**Figure legends**

**
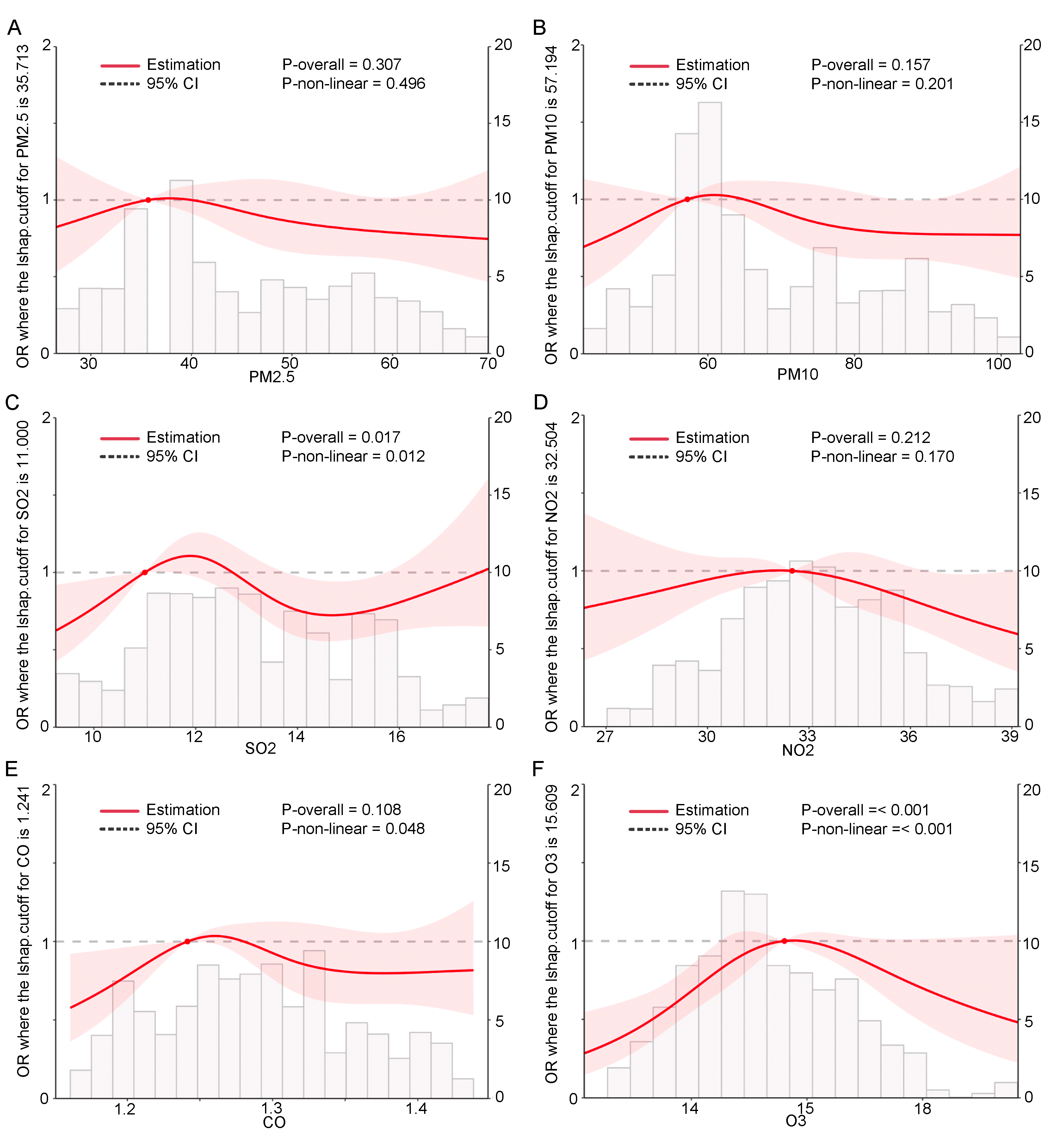
**

**Figure S1.** The relationship between six pollutants in the first trimester and the OR of placenta accreta without previa in twin pregnancies was fitted using restricted cubic splines. The reference level for OR was the median pollution level, and the baseline was set at Y = 1. The curves were adjusted for maternal age, pre-pregnancy body mass index (BMI, calculated as weight in kilograms divided by height in meters squared), primigravida and nulliparity status, ART use, family history (hypertension, hyperglycemia), presence of a scarred uterus and uterine fibroids, chorionicity (DCDA, non-DCDA), conception season (spring, summer, autumn, or winter), and Tmean . P-overall tests the overall significance of the relationship (linear and nonlinear); P-nonlinear tests whether the relationship is nonlinear. The shaded area represents the 95% confidence interval.


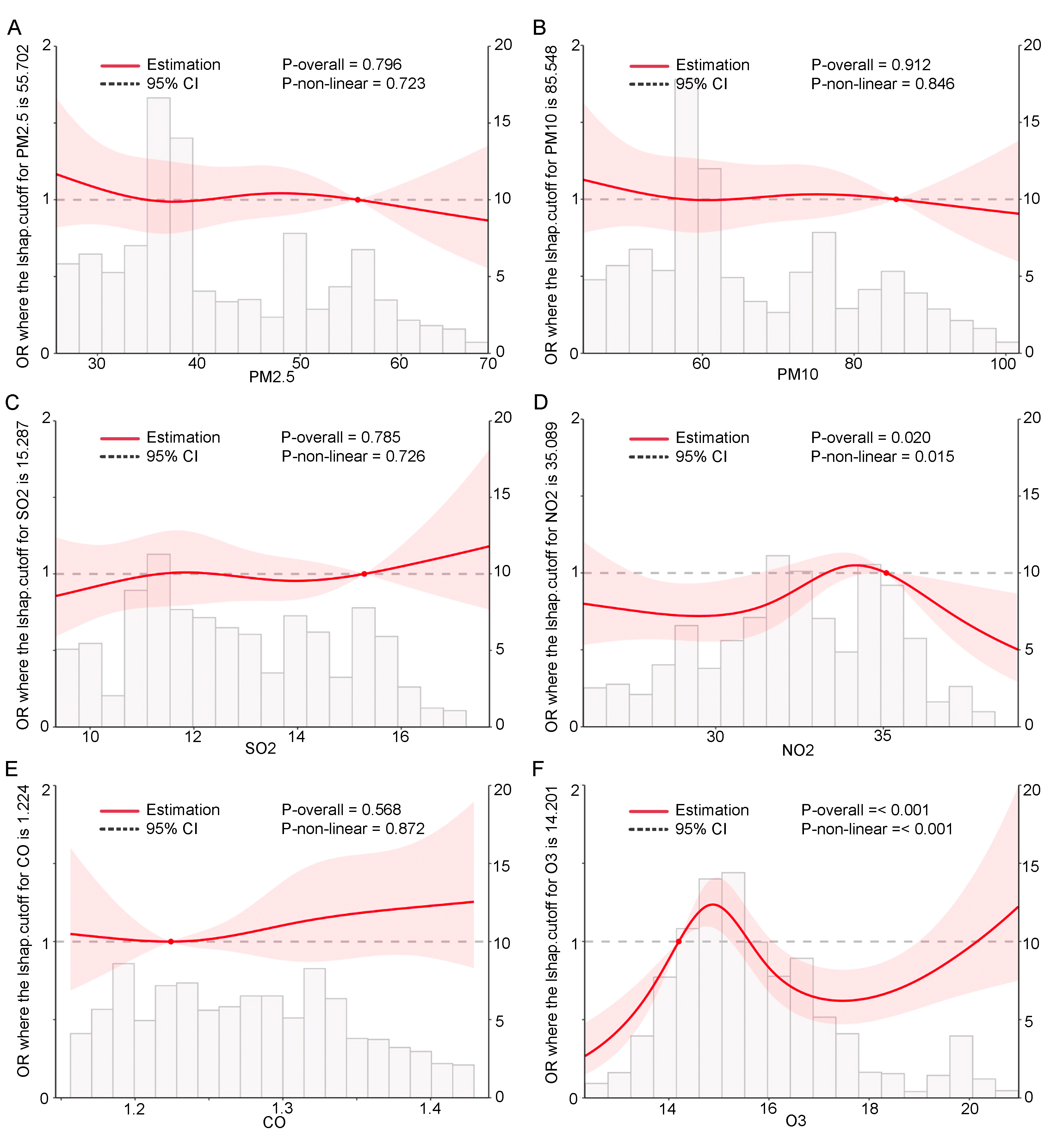


**Figure S2.** The relationship between six pollutants in the second trimester and the OR)of placenta accreta without previa in twin pregnancies was fitted using restricted cubic splines. The reference level for OR was set at the median pollution level, with the baseline at Y = 1. These curves were adjusted for maternal age, pre-pregnancy body mass index (BMI, calculated as weight in kilograms divided by height in meters squared), primigravida and nulliparity status, ART use, family history (hypertension, hyperglycemia), presence of a scarred uterus and uterine fibroids, chorionicity (dichorionic diamniotic [DCDA], non-DCDA), conception season (spring, summer, autumn, or winter), and Tmean . P-overall tests the overall significance of the relationship (linear and nonlinear); P-nonlinear tests whether the relationship is nonlinear. The shaded area represents the 95% confidence interval.
